# AHL-mediated quorum sensing drives plastisphere formation and elevates pathogenic potential

**DOI:** 10.1093/ismejo/wrag066

**Published:** 2026-03-24

**Authors:** Jie Wang, Lijia Lu, Yuanze Sun, Lauren F Messer, Mochen Wu, Zhuoran Duan, Jia Shi, Yuyi Yang, Changchao Li, Yanping Mao, Dong Zhu, Matthias C Rillig, Xiaoping Wang

**Affiliations:** College of Resources and Environmental Sciences, China Agricultural University, Haidian District, Beijing 100193, China; College of Resources and Environmental Sciences, China Agricultural University, Haidian District, Beijing 100193, China; Institute of Biology, Freie Universität Berlin, Altensteinstraße 6, 14195 Berlin, Berlin, Germany; Organisms and Ecosystems, Earlham Institute, Norwich, Norfolk NR4 7UZ, United Kingdom; College of Resources and Environmental Sciences, China Agricultural University, Haidian District, Beijing 100193, China; College of Resources and Environmental Sciences, China Agricultural University, Haidian District, Beijing 100193, China; College of Resources and Environmental Sciences, China Agricultural University, Haidian District, Beijing 100193, China; Research Center for Environmental Ecology and Engineering, School of Environmental Ecology and Biological Engineering, Wuhan Institute of Technology, Wuhan 430205, Hubei, China; Department of Civil and Environmental Engineering, The Hong Kong Polytechnic University, Hung Hom, Kowloon, Hong Kong 999077, Hong Kong, China; College of Chemistry and Environmental Engineering, Shenzhen University, Shenzhen, Guangdong 518071, China; Key Laboratory of Urban Environment and Health, Ningbo Urban Environment Observation and Research Station, Institute of Urban Environment, Chinese Academy of Sciences, Xiamen, Fujian 361021, China; Institute of Biology, Freie Universität Berlin, Altensteinstraße 6, 14195 Berlin, Berlin, Germany; College of Resources and Environmental Sciences, China Agricultural University, Haidian District, Beijing 100193, China

**Keywords:** plastisphere, quorum sensing, acyl-homoserine lactone, quorum quenching, multiomics analyses

## Abstract

The biofilm colonizing plastic debris, termed “the plastisphere,” is of growing global concern due to escalating plastic pollution. However, the biological mechanisms underpinning plastisphere formation remain poorly understood. Here, we analyzed public global metagenomes, revealing a significant enrichment of genes associated with quorum sensing (QS) and biofilm formation, with a pronounced signal for acyl-homoserine lactone (AHL) QS. Using controlled microfluidic and tubular column experiments, we further demonstrate that exogenous AHL actively promotes plastisphere formation, biomass accumulation, and extracellular polymeric substance production on microplastics, whereas a quorum-quenching agent (AHL acylase) effectively inhibits these processes. Multi-omics analyses revealed that AHLs can transcriptionally activate genes involved in adhesion, motility, chemotaxis, and matrix production, fundamentally reshaping community structure, restructuring inferred microbial interaction networks, and driving community assembly toward stronger deterministic selection. AHL stimulation also increased the relative abundance and expression of pathogen-associated and virulence-related functions, suggesting an elevated virulence potential within the plastisphere under QS-promoting conditions. Together, our findings establish AHL-mediated QS as a central driver of plastisphere assembly and a key determinant of risk profile, highlighting its critical role in understanding and potentially mitigating the growing environmental and health hazards associated with microplastic pollution.

## Introduction

The widespread utility of plastics, although driving modern life and industry, has created a persistent and escalating global challenge in the form of plastic waste [1]. By 2050, global plastic waste is projected to reach a staggering 12 000 million tons [2]. This plastic can undergo progressive fragmentation into microplastics (diameter < 5 mm) due to factors like ultraviolet radiation, physical abrasion, and biological activity [3, 4, 5]. Microplastics are omnipresent across Earth’s biomes, from Mt. Everest to the Mariana Trench [6, 7]. As pervasively persistent pollutants of anthropogenic origin, microplastics have galvanized international attention and attracted rapidly growing research into their risks to human and environmental health.

Beyond their direct environmental and health concerns, microplastics offer selective artificial surfaces for microbial colonization and biofilm growth, creating a unique ecological niche known as the “plastisphere” [8, 9, 10]. Mounting evidence suggests that plastisphere microbial communities exhibit significant differences compared to those found on surrounding natural substrates [11, 12]. The plastisphere can be a reservoir of potential pathogens, antibiotic resistance genes (ARGs), and virulence factors (VFs) [13, 14]. Considering the substantial microbial biomass within the plastisphere and the environmental mobility of microplastics, these artificial biofilm communities pose substantial risks to biological safety and human health [15]. Despite this growing concern, the mechanistic underpinning of how biofilm establishes and develops on microplastic surfaces remains incompletely understood [16], with previous research largely focusing on external factors (e.g. environmental conditions or polymer types) and early adhesion/extracellular polymeric substance (EPS) processes rather than regulatory signaling mechanisms.

Biofilm formation is an intricate, multi-stage process that begins with initial attachment, followed by microcolony formation and extracellular matrix production, and the process requires population-wide coordination of individual cells [17]. To orchestrate these collective behaviors, microorganisms employ a cell-to-cell communication process called quorum sensing (QS), a mechanism that can activate the expression of related genes through signaling molecules and trigger coordinated behaviors, including biofilm formation [18, 19, 20]. QS can determine microbial community structure by shaping microbial interactions to respond and adapt to dynamic environments [21, 22]. Given the critical links between QS and biofilm formation, we hypothesized that plastisphere establishment and growth are, at least in part, QS-dependent.

Among the diverse QS systems used by microbes, including acyl-homoserine lactones (AHLs), autoinducer-2 (AI-2), autoinducing peptides (AIPs), quinolone-like 2-heptyl-3-hydroxy-4-quinolone (PQS), diffusible signal factor (DSF), and cyclic dimeric (3–5) GMP (c-di-GMP), AHLs are primary QS signals for many Gram-negative taxa and are repeatedly linked to biofilm formation and EPS production [23–26]. Because many plastisphere-associated bacteria belong to AHL-using lineages, e.g. Pseudomonadota, and plastics may provide hydrophobic sorptive surfaces that can retain lipophilic signals, we hypothesized that AHLs can actively stimulate QS processes and facilitate plastisphere formation on microplastics, whereas QS inhibitors (e.g. AHL acylases) display the opposing effects (quorum quenching, QQ). To date, whether QS signaling processes occur within the unique architectural constraints of the plastisphere, and how they impact community structure and functional characteristics, remains a substantial knowledge gap. Studies specifically addressing the regulatory roles of QS inhibitors in the plastisphere are currently lacking.

**Figure 1:**
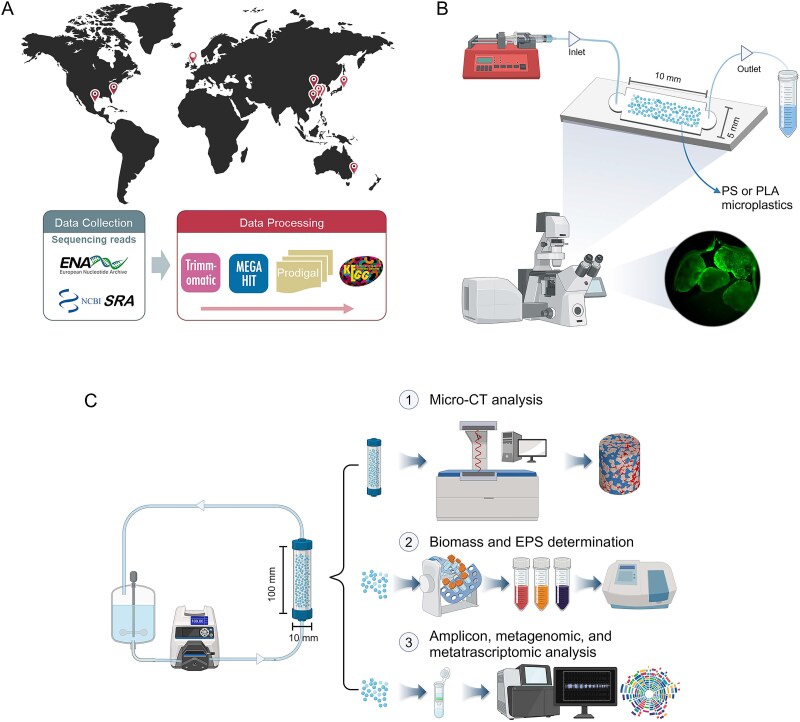
Overview of the study design and experimental setups. (A) Sources of the plastisphere and associated ambient water samples that were analyzed in this study from scientific publications and public databases. (B) Schematic illustration of the microfluidic device used for visualizing plastisphere formation dynamics on microplastics under controlled flow and treatment conditions. (C) Schematic diagram of the tubular column incubation system for larger-scale plastisphere cultivation, showing the setup for continuous flow culture and subsequent analysis.

Here, we analyzed publicly available plastisphere metagenomes from global sources to investigate the relationship between QS processes and biofilm formation. Subsequently, microfluidic incubation and tubular column systems were developed to monitor biofilm colonization on microplastics *in situ* using both 2D and 3D perspectives (Fig. 1) via confocal laser scanning microscopy and X-ray microcomputed tomography. We then employed 16S ribosomal Ribonucleic Acid (rRNA) gene amplicon sequencing, metagenomics, and metatranscriptomics to elucidate the underlying QS mechanisms of plastisphere formation. Our research provides valuable insights into how QS signaling coordinates community behaviors and associated functions fundamental to plastisphere formation.

## Materials and methods

### Metadata collection and data processing

Publicly available metagenomic data related to the plastisphere were compiled from scientific publications, the National Center for Biotechnology Information Bioproject database, and the European Nucleotide Archive database (Fig. 1A). The literature/repository search was conducted up to December 2024, and the compiled dataset spans multiple publication years (2019–24). For each study, extracted available metadata (e.g. ecosystem type, sampling location, and polymer type) are summarized in Supplementary Table S1. Data filtering criteria included: (i) plastisphere samples were collected or incubated in freshwater or seawater ecosystems; (ii) raw sequence data for both the plastisphere and corresponding water samples were available to allow for comparative analysis of QS potential; and (iii) studies that were exclusively focused on plastisphere data were excluded. Applying these criteria yielded a dataset of 166 plastisphere and 54 ambient water metagenomes (Supplementary Table S1).

Raw sequencing data were quality-controlled using fastp (v0.24.0), followed by trimming and quality filtering with Trimmomatic (v0.40) [27]. Cleaned reads were assembled using Megahit (v1.2.9), and assembled contigs with length > 500 bp were retained [28]. Open reading frames (ORFs) were predicted from the contigs using Prodigal (v2.6.3) and clustered into a nonredundant gene catalog using CD-HIT (v4.8.1) with 95% identify and 90% coverage [29]. ORFs were annotated via BLASTX against the Kyoto Encyclopedia of Genes and Genomes (KEGG) database. Gene abundances were quantified as transcripts per kilobase million (TPM) using Salmon (v1.10.1) [30]. Based on the types of signal molecules, the quorum-sensing system was divided into six main categories: acyl-homoserine lactones (AHLs), autoinducing peptides (AIPs), autoinducer-2 (AI-2), quinolone-like 2-heptyl-3-hydroxy-4-quinolone (PQS), diffusible signal factor (DSF), and cyclic dimeric (3–5) GMP (c-di-GMP).

Assembled contigs were further binned to metagenome-assembled genomes (MAGs) using an ensemble binning approach with metaBAT2 (v2.17), MaxBin2 (v2.2.7), SemiBin2 (v2.2.0), and VAMB (v5.0.3) to maximize genome recovery. Binning results were consolidated by selecting a nonredundant set of MAGs using DAS Tool (v1.1.4) based on a score threshold of 0.7 and deduplicated using dRep (v3.4.3). MAG quality was assessed with CheckM2 (v1.0.1), and good-quality MAGs with a minimum completeness of 50% and maximum contamination of 10% were retained for further analysis. A total of 1979 MAGs were obtained, and 915 MAGs can be classified as high quality (i.e. completeness >90%, contamination <5%). MAG abundance was quantified using CoverM (v0.7.0), and taxonomic assignments were performed using GTDB-TK (v2.1.1) with the GTDB r207 database. A phylogenetic tree of the MAGs was plotted with the R packages ape (v5.7.1), ggtree (v3.11.0), and tidytree (v0.4.5). The presence of AHL-related genes within MAGs was annotated via BLASTX against the QS-related protein (QSP) database [31].

### Experimental chemicals

Two typical and extensively applied microplastics, including conventional polystyrene (PS) and biodegradable polylactic acid (PLA) [32, 33], were purchased from Aladdin (Shanghai, China) and NatureWorks (Minneapolis, MN, USA), respectively. Microplastics were cleaned with ultrapure water, air-dried in a fume hood, and stored at 4°C. The standard QS signaling molecule *N*-(Ketocaproyl)-DL-homoserine lactone (3OC6-HSL) was purchased from Sigma-Aldrich (USA). A 10 mg/l stock solution was prepared by dissolving 3OC6-HSL powder in methanol (High-Performance Liquid Chromatography grade, 99.9%) and stored in tightly sealed vials at −20°C. The final concentration of 3OC6-HSL in QS treatments was 10 μg/l (~50 nM), selected based on established QS response thresholds for initial adhesion/biofilm development in wastewater biofilm [34] and within ranges used to evaluate AHL effects on bacterial adhesion onto plastics in aquatic matrices [35], although acknowledging that bulk-water AHL concentrations reported in engineered/environmental waters are often lower (nanograms per liter scale) [36, 37] and that local concentrations at biofilm–surface interfaces may be higher due to retention in biofilms/sediments [38] and adsorption onto plastics [35]. To further justify this specific dose, we evaluated the EPS properties on microplastics exposed to varying concentrations of 3OC6-HSL. We observed that the ratio of protein to polysaccharide was significantly higher at the 10 μg/l concentration, indicating a distinct, QS-driven structural shift in the EPS matrix that supports robust biofilm formation (Supplementary Fig. S19). To control for the carrier solvent, equal volumes of methanol were added to all treatments (final methanol 0.1% v/v). AHL acylase (CAS 9012-37-7, specific activity ≥30 000 unit/g), used as a QQ enzyme, was purchased from Aladdin (Shanghai, China).

### Plastisphere formation in microfluidic chips

Microfluidic chips with a chamber size of 10 mm (length) × 5 mm (width) × 200 μm (depth) were fabricated from polydimethylsiloxane (PDMS) using soft lithography with the assistance of Wuhan Jianmi Intelligent Control Technology Co., Ltd. (Fig. 1B). Each chamber was packed with a monolayer of PS or PLA microplastic particles (200 ± 10 μm diameter) and bonded firmly to glass slides. The chips were integrated into a continuous-flow system with a single inlet and outlet, driven by a syringe pump. Prior to use, all microfluidic devices were disinfected using 75% ethanol for 15 min, followed by thorough rinsing with sterile deionized water.

Filtered effluent (1.2 μm glass fiber filter membrane) from the Xiaojiahe Wastewater Treatment Plant served as the incubation media, supplied at a constant flow rate of 1 μl/min. Incubation was performed at 25°C in the dark. Four treatment conditions were established: control (CK; no additions), QS (10 μg/l 3OC6-HSL added to medium), QQ (AHL acylase added to medium), and QSQQ (both 3OC6-HSL and AHL acylase added). To capture early-stage colonization dynamics under imaging-compatible conditions, microfluidic incubations were performed for 48 h. For each polymer × treatment, three independent microfluidic chips were run. After incubation, plastispheres within microfluidic chambers were stained using the fluorescent dye SYTO 9 for 30 min and subsequently imaged using a confocal microscope (FV3000, Olympus) at 10× magnification. The entire area of each microfluidic chamber was scanned, acquiring images of 1024 × 1024 pixels. Mean fluorescence intensity of the plastisphere biofilm was quantified using ImageJ software as a proxy for biomass.

### Tubular column incubation for plastisphere

Plastisphere formation was also investigated in a larger-scale, continuous-flow tubular column culture system consisting of a glass column and a peristaltic pump (Fig. 1C). Glass columns (100 mm length, 10 mm diameter) were packed with PS or PLA microplastic grains, each averaging ~1.5 mm in diameter. The incubation medium was identical to that used in the microfluidic experiments, supplied at a constant flow rate of 5 ml/min. Four treatment groups (CK, QS, QQ, and QSQQ) were evaluated. For each polymer × treatment, six independent columns were run. Because larger columns require longer times to develop mature, 3D biofilms and to yield sufficient biomass for micro-CT imaging and multi-omics, tubular columns were incubated for 10 days. After incubation, *in situ* 3D biofilm structures on the microplastics were acquired using X-ray micro-computed tomography (micro-CT, Phoenix Vtomex S240, Waygate Technologies). The resulting projection images were then reconstructed into 3D volumetric data using Avizo 9.3.0 software (FEI Hillsboro, Or, USA). This allowed for the differentiation and quantification of both microplastic particle and biofilm volumes.

### Plastisphere biomass and extracellular polymeric substance quantification

Plastisphere biomass was quantified using a crystal violet assay [39]. Briefly, 20 microplastic pellets were sampled from each tubular column, gently rinsed three times with sterile phosphate buffer solution (PBS), and air-dried for 45 min in a sterile Petri dish. Pellets were stained with 1% crystal violet (w/v) for 45 min, washed three times with sterile PBS, and air-dried again for 45 min. Stained pellets were transferred into a 15 ml centrifuge tube, and 1 ml of 95% ethanol (v/v) was added to elute the dye. The optical density of the resulting ethanol solution, directly proportional to biomass, was measured at 595 nm using a Ultraviolet-Visible (UV-Vis) spectrophotometer (UV-1900i, Shimadzu, Japan). Protein and polysaccharide content in extracellular polymeric substance (EPS) of plastisphere were determined following the established method [40, 41]. Briefly, twenty microplastic pellets were transferred into a 15 ml centrifuge tube, and EPS was extracted using 10 ml of 2% (w/v) Ethylenediaminetetraacetic acid disodium salt solution, stirred for 3 h at 4°C. The supernatant was collected by centrifugation (5000 *g*, 4°C, 10 min) and filtered through a 0.22 μm polycarbonate membrane. Protein concentration was measured at 562 nm using a BCA (bicinchoninic acid) protein assay with bovine serum albumin as standard. Polysaccharide concentration was determined at 490 nm using the phenol-sulfuric acid colorimetric method with glucose as standard. For each metric, the mean value per column (*n* = 6 columns per polymer × treatment) was used for statistical comparisons.

### Multi-omics sequencing and analysis

Approximately 200 microplastic pellets from each tubular column treatment were used for plastisphere DNA extraction using the DNeasy PowerSoil Kit (Qiagen, Shanghai, China) for 16S rRNA gene sequencing (*n* = 6 per polymer × treatment). The V3–V4 region of the 16S rRNA gene was amplified using universal primers 338F (5′-ACTCCTACGGGAGGCAGCAG-3′) and 806R (5′-GGACTACHVGGGTWTCTAAT-3′) [42]. Polymerase chain reaction (PCR) was performed in a total volume of 25 μl reaction volume, containing 1 μl diluted DNA, 1 μl each PCR primer, 12.5 μl PCR buffer including 0.5 unit of high-fidelity Accuprime Taq DNA polymerase (Invitrogen, Carlsbad, CA, USA), and 9.5 μl nucleic acid–free water. The PCR program consisted of 3-min initial DNA denaturation at 95°C, 27 cycles at 95°C for 30 s, 55°C for 30 s, and 72°C for 30 s and 10-min elongation at 72°C.

Amplicons were purified and sequenced on a MiSeq System (Illumina) using 2 × 300 bp paired-end reads. Raw sequencing data were processed following the UNOISE pipeline. Forward- and reverse-paired reads were merged, and reads with a quality score < 30 or length < 300 bp were filtered. Singletons were removed, and sequences were denoised using UNOISE3 at 100% similarity to obtain zero-radius operational taxonomic units (ZOTUs) [43]. Taxonomic assignment was performed using the RDP classifier with a threshold of 0.8 against the SILVA reference database (version 138) [44]. Community diversity, composition structure, species interaction, network analysis, and community assembly processes were analyzed as detailed in the Supplementary Information.

For metagenomic and metatranscriptomic sequencing, ~500 microplastic pellets per treatment were collected for DNA and RNA extraction (*n* = 4 per polymer × treatment). Total DNA was extracted using the DNeasy PowerSoil Kit (Qiagen, Shanghai, China) and further purified using the Genomic DNA Clean & Concentrator kit (Zymo Research). Total RNA was extracted using the RNeasy PowerSoil Total RNA Kit (Qiagen, Shanghai, China) and treated with DNase using the TURBO DNA-free kit (ThermoFisher Scientific). DNA sequencing was performed on a NovaSeq X Plus System (Illumina), generating ~12 Gbp of 2 × 150 bp paired-end reads per sample. Extracted RNA was converted to cDNA and sequenced to obtain metatranscriptomic data.

Metagenomic data analyses followed the same workflow described previously for the public datasets. For metatranscriptomic analyses, raw reads were quality-controlled using fastp (v0.24.0), and rRNA reads were removed using SortMeRNA (v2.1b) [45]. Clean reads were assembled using Trinity (v2.2.0) with a minimum contig length of 300 bp [46]. ORFs were predicted using Prodigal (v2.6.3) and clustered into a nonredundant gene catalog using CD-HIT (v4.8.1) with 95% identify and 90% coverage [29, 47]. ORFs were annotated via BLASTX against the KEGG and the Pathogen Host Interactions (PHI) databases. Gene expression levels were quantified as TPM using Salmon (v1.10.1) [30]. Using the raw functional profiling abundances calculated for metagenomes and metatranscriptomes above, we calculated gene-normalized transcript abundances using the ratio of transcript abundances (RNA) to predicted gene abundances (DNA) for gene expression analysis.

### Statistical analysis

We tested whether QS and biofilm formation differed between plastisphere and water samples using Wilcoxon rank-sum tests for the public metagenomes. Kruskal–Wallis test with Dunn’s multiple comparisons was used to determine the differences in biofilm biomass, protein, and polysaccharide concentrations among the different incubated treatments. Statistical significance was defined as *P* < .05. All the statistical analyses were performed in R version 4.3.2.

## Results

### Enrichment of acyl-homoserine lactone quorum-sensing and biofilm formation genes in the plastisphere

**Figure 2:**
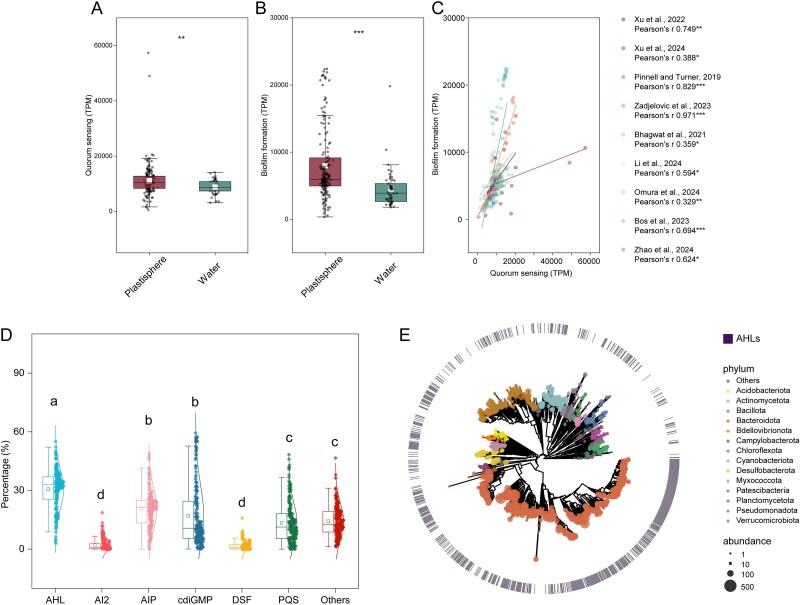
Enrichment of quorum sensing and biofilm formation genes in the plastisphere revealed by global metagenome analysis. (A) Abundance of genes associated with the overall quorum-sensing pathway in the plastisphere versus water samples. (B) Abundance of genes associated with the biofilm formation pathway in the plastisphere and water samples. Both (A and B) show significantly higher abundance in plastispheres (Wilcoxon rank-sum test, *P* < .01). (C) Pearson correlation between the abundances of quorum sensing and biofilm formation within plastisphere communities across individual studies. (D) Relative abundance of functional genes associated with different types of quorum-sensing signals across all plastisphere samples. (E) Maximum likelihood phylogenetic tree of the metagenome-assembled genomes (MAGs) obtained from plastisphere metagenomes. The size of the nodes shows the average abundance of the different MAGs, and the presence of AHL-related genes in MAGs is indicated.

Following rigorous data filtering, we compiled 166 plastisphere metagenomes and 53 metagenomes of associated planktonic microorganisms from nine independent studies in which plastisphere and ambient water samples were coreported from the same sampling campaigns (Fig. 1A and Supplementary Table S1). Plastisphere samples exhibited significantly higher abundances (*P* < .01) of genes related to QS and biofilm formation (Fig. 2A and B). The mean ± standard error for the abundance of the QS gene was 11 090 ± 472 TPM in plastisphere samples versus 8820 ± 358 TPM in planktonic communities, whereas biofilm formation gene abundance was 7980 ± 417 TPM versus 4500 ± 414 TPM, respectively. Within each of the nine studies, a positive correlation was observed between the abundances of biofilm formation and QS genes (Fig. 2C), with a Pearson correlation coefficient *r* ranging from 0.329 to 0.971 (*P* < .05), strongly suggesting a consistent link between QS potential and plastisphere biofilm functions across studies.

Further classification of functional QS genes into six main categories (acyl-homoserine lactones [AHLs], autoinducing peptides [AIPs], and autoinducer-2 [AI-2], quinolone-like 2-heptyl-3-hydroxy-4-quinolone [PQS], diffusible signal factor [DSF], and cyclic dimeric (3–5) GMP [c-di-GMP]) revealed a significantly higher abundance of genes specifically involved in AHL QS processes across the plastisphere samples (Fig. 2D). To link this QS potential to specific taxa, we assembled metagenomic reads into 1979 medium- to high-quality metagenomic-assembled genomes (MAGs), including 915 classified as high quality (Supplementary Fig. S1). Taxonomic analysis revealed dominance by Pseudomonadota (*n* = 934, 60.1% relative abundance), Bacteroidota (*n* = 297, 12.7%), and Actinomycetota (*n* = 152, 9.6%) across plastisphere samples, alongside a substantial fraction of previously uncharacterized taxa at various taxonomic ranks (Supplementary Fig. S2). Genes involved in AHL biosynthesis and reception were widely distributed among these dominant plastisphere MAGs (Fig. 2E), indicating that key members of the plastisphere community are genetically equipped for AHL-mediated communication, likely contributing significantly to biofilm development on plastics.

### Evidence for acyl-homoserine lactone quorum sensing facilitating plastisphere formation

**Figure 3:**
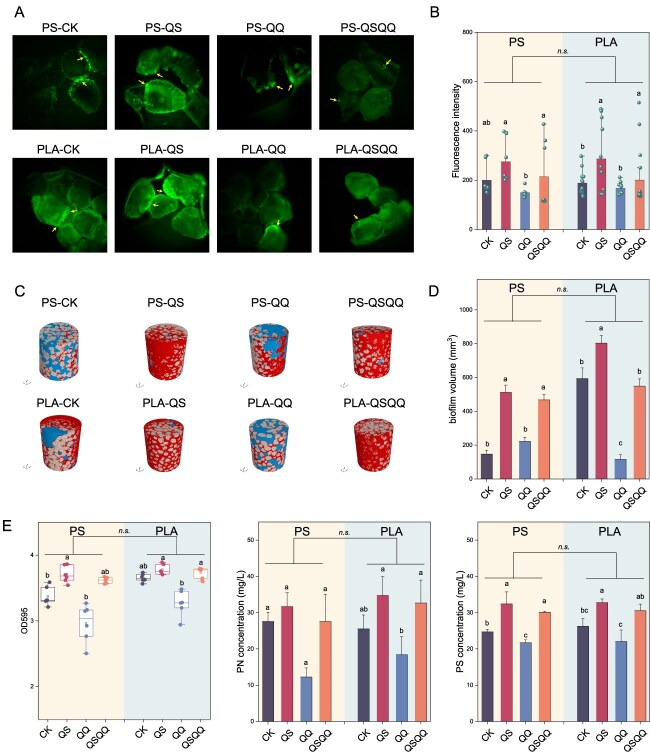
AHL quorum sensing promotes plastisphere formation and biomass. (A) Confocal microscopy images showing the architecture of plastisphere biofilms (stained green with SYTO 9) on microplastic particles in microfluidic chips under different quorum sensing and quenching manipulation. Control (CK), quorum sensing (QS), quorum quenching (QQ), and combined QS and QQ (QSQQ). (B) Quantification of plastisphere biofilm biomass based on mean fluorescence intensity from confocal images using ImageJ software. (C) Three-dimensional rendering from micro-CT imaging showing plastisphere biofilm (red) colonizing microplastic particles (gray) and pore space (blue) in tubular columns after 10 days. (D) Quantification of plastisphere biofilm volume relative to total microplastic volume from micro-CT data using Avizo software. (E) Total biofilm biomass (crystal violet assay), extracellular polysaccharide, and protein content measured in plastispheres from tubular column experiments. In (B, D, and E), different letters indicate statistically significant differences between treatments (Kruskal–Wallis test with Dunn’s multiple comparisons, *P* < .05). Error bars show the standard deviation of the mean.

To directly visualize and quantitatively assess the influence of AHLs and their inhibition on plastisphere biofilm formation dynamics, we utilized a microfluidic chip platform with embedded exogenous microplastic particles (Fig. 1B), enabling controlled direct observation of plastisphere dynamics under varying QS and QQ manipulations. After 48 h of growth, substantial biofilm colonization was observed across the surfaces of microplastic particles under QS treatments, whereas the QQ treatment resulted in significantly less coverage, with biofilm primarily limited to particle edges and corners (Fig. 3A). Quantitative image analysis of fluorescence intensity, serving as a proxy for biofilm biomass, revealed the highest levels in the QS treatment, followed by treatments combining QS and QQ (QSQQ), control (CK), and the lowest levels in the QQ treatment (Fig. 3B). No significant differences in fluorescence intensity were observed when comparing the PS and PLA plastisphere in this microfluidic system.

To further assess the chemical and genomic regulation of QS and QQ manipulation on plastisphere dynamics, we employed a larger-scale tubular column incubation system (Fig. 1C). After 10 days, micro-CT imaging provided *in situ* 3D reconstructions, showing extensive biofilm formation fully encasing microplastic grains in the QS treatment, in stark contrast to the restricted, patchy colonization observed under QQ treatment (Fig. 3C). Quantitative volume analysis confirmed that the plastisphere volume was highest under QS stimulation and lowest under QQ inhibition (Fig. 3D). Consistently, plastisphere volume did not differ significantly between PS and PLA microplastics, suggesting that the impact of QS and QQ on biofilm volume is largely independent of these polymer types.

Consistent with imaging results, biochemical analysis of plastispheres demonstrated that QS treatment significantly stimulated total biofilm biomass, as well as extracellular polysaccharide and protein content, compared to controls, whereas QQ treatment caused significant decreases (Fig. 3E). Specifically for the PLA plastisphere, QS treatment resulted in polysaccharide and protein concentrations of 32.7 ± 2.1 and 34.7 ± 7.6 mg/l, respectively, significantly higher than control levels (26.2 ± 4.2 and 25.5 ± 5.1 mg/l; *P* < .05). Conversely, QQ treatment yielded significantly lower concentrations (22.0 ± 3.1 and 18.4 ± 5.8 mg/l; *P* < .05). Similar trends were observed for conventional PS microplastics. Together, these imaging and biochemical results demonstrate that the AHL signal (3OC6-HSL) promotes plastisphere formation and biomass accumulation under our experimental conditions, whereas the AHL acylase quencher effectively suppresses these responses.

### Distinct microbial assemblages induced by acyl-homoserine lactone quorum sensing

**Figure 4:**
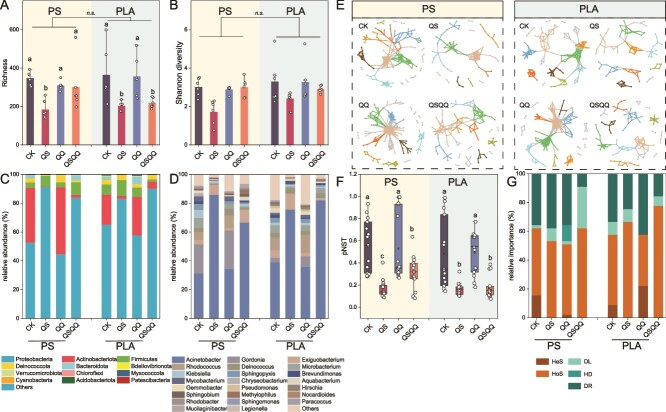
AHL quorum sensing fundamentally reshapes plastisphere community structure and assembly. (A) Bacterial richness and (B) Shannon Index of plastisphere communities from tubular column experiments under different treatments. (C) Relative abundance of bacterial phyla and (D) Bacterial genera across different treatments. (E) Co-occurrence interaction networks inferred for bacterial communities under each treatment, illustrating relationships between taxa. (F) Normalized stochasticity ratios (pNST) reflecting the relative influence of deterministic versus stochastic processes on community assembly. (G) Relative importance of different ecological assembly processes inferred using the iCAMP model. HeS, heterogeneous selection; HoS, homogeneous selection; DL, dispersal limitation; HD, homogenizing dispersal; DR, drift. In (A, B, and F), different letters indicate statistically significant differences between treatments (Kruskal–Wallis test with Dunn’s multiple comparisons, *P* < .05). Error bars show the standard deviation of the mean.

Analysis of 16S rRNA gene sequencing data revealed significant alterations in microbial community structure in response to QS and QQ manipulation. Although alpha-diversity metrics (Shannon Index and richness) were generally higher in bulk water compared to all plastisphere samples (Supplementary Fig. S3), within the plastisphere, both diversity and richness were lowest in the plastispheres treated with the QS molecule (Fig. 4A and B). Beta-diversity analysis using Bray–Curtis dissimilarities demonstrated a clear and significant clustering of plastisphere communities based on treatment, distinctly separating samples supplemented with the QS molecule from those without (Supplementary Fig. S4). Compared to bulk water communities, QS- and QSQQ-treated plastispheres exhibited lower similarity, whereas CK and QQ treatments maintained higher similarity (Supplementary Fig. S4). This distinct compositional shift indicates that QS processes play a significant role in shaping microbial recruitment and community structure within the plastisphere. At the phylum level, Pseudomonadota dominated QS- and QSQQ-treated plastispheres (83.2%–91.3%), contrasting with the prevalence of Actinomycetota in CK and QQ treatments (Fig. 4C). At the genus level (Fig. 4D), *Acinetobacter* exhibited significantly higher abundance in QS- and QSQQ-treated plastisphere than those in CK and QQ treatments (66.5%–85.9% vs. 34.5%–35.9%, *P* < .05). The type of plastic polymer did not significantly influence overall bacterial community structure (Supplementary Fig. S4, *R*^2^ = 0.066, *P* > .05).

To infer the difference in microbial interactions and community organization under QS and QQ manipulation, we constructed co-occurrence networks. Networks derived from QS- and QSQQ-treated plastispheres were less complex than those from CK and QQ treatments (Fig. 4E and Supplementary Table S2), exhibiting significantly smaller sizes (76–110 vs. 128–175 nodes, Supplementary Table S2), lower connectivity (112–162 vs. 270–612 links), lower average connectivity (2.69–3.45 vs. 4.22–6.99), and lower clustering coefficient (0.081–0.192 vs. 0.163–0.296). A higher proportion of negative links in QS- and QSQQ-treated networks suggested intensified competitive interactions among microorganisms, particularly pronounced in the conventional PS plastisphere (Supplementary Table S2). Higher modularity values (0.603–0.809) in QS/QSQQ networks compared to CK/QQ indicated a greater extent of community compartmentalization or niche differentiation. Stability analysis through random node removal demonstrated that networks formed under QS/QSQQ conditions were consistently less robust than controls (Supplementary Fig. S5). In addition to the topological properties, analysis of dominant nodes and edges further revealed shifts in key taxa and interactions structuring the networks across treatments (Supplementary Figs S6–S8). Although Gammaproteobacteria, Alphaproteobacteria, and Bacteroidia were major classes in nodes across all networks, QS and QSQQ treatments decreased the proportion of Alphaproteobacteria nodes and increased the prevalence of edges linking Gammaproteobacteria and Bacteroidia.

Analysis of bacterial niche breadth at the community level revealed significantly narrower niches for plastisphere communities under QS treatment (Supplementary Fig. S9). To quantify the relative influence of stochastic versus deterministic processes shaping community assembly, we calculated normalized stochasticity ratios based on phylogenetic normalized stochasticity ratio (pNST). The values were significantly lower in QS- and QSQQ-treated PS plastisphere (0.171 and 0.371) compared to CK and QQ treatments (0.548 and 0.532) (Fig. 4F), indicating a shift toward more deterministic assembly under QS stimulation, a trend also observed for PLA plastisphere. Inferring community assembly mechanisms by the phylogenetic-bin-based null model (iCAMP) was further used to estimate the contribution of stochastic and deterministic processes. Although homogeneous selection and drift were the dominant drivers of community assembly across all treatments, the contribution of homogeneous selection was higher in QS- and QSQQ-treated plastispheres (Fig. 4G). Combined, these findings strongly suggest that the QS molecule exerts a potent deterministic selective pressure under the experimental conditions, favoring the recruitment and dominance of specific species highly adapted to the biofilm lifestyle facilitated by QS within the plastisphere.

### Acyl-homoserine lactone molecule activates quorum sensing and subsequent biofilm formation on microplastics

To investigate the molecular mechanisms by which the AHL signal and AHL acylase regulate plastisphere development, we performed metagenomic and metatranscriptomic analyses on the tubular column samples. Procrustes analysis and Mantel tests revealed strong correlations among community structure (16S rRNA gene), predicted functional potential (metagenomics), and active gene expression (metatranscriptomics) (Supplementary Fig. S10), indicating that different omics approaches provided a cohesive view of the plastisphere ecosystem. Similar to the 16S rRNA gene sequencing results, Pseudomonadota dominated QS- and QSQQ-treated plastispheres. Metagenomic analysis showed higher gene abundances for overall QS pathways in CK- and QQ-treated plastispheres, which exhibited less biofilm (Supplementary Fig. S11A), likely reflecting the presence of a broader set of taxa carrying diverse QS-related genes under these more diverse community states. In contrast, metatranscriptomic profiles revealed that the active expression of QS genes aligned with observed biofilm levels, suggesting that AHL addition primarily increases QS activity, rather than necessarily increasing total genomic QS gene content, by simulating signal reception and downstream transcriptional responses (Supplementary Fig. S11B).

**Figure 5:**
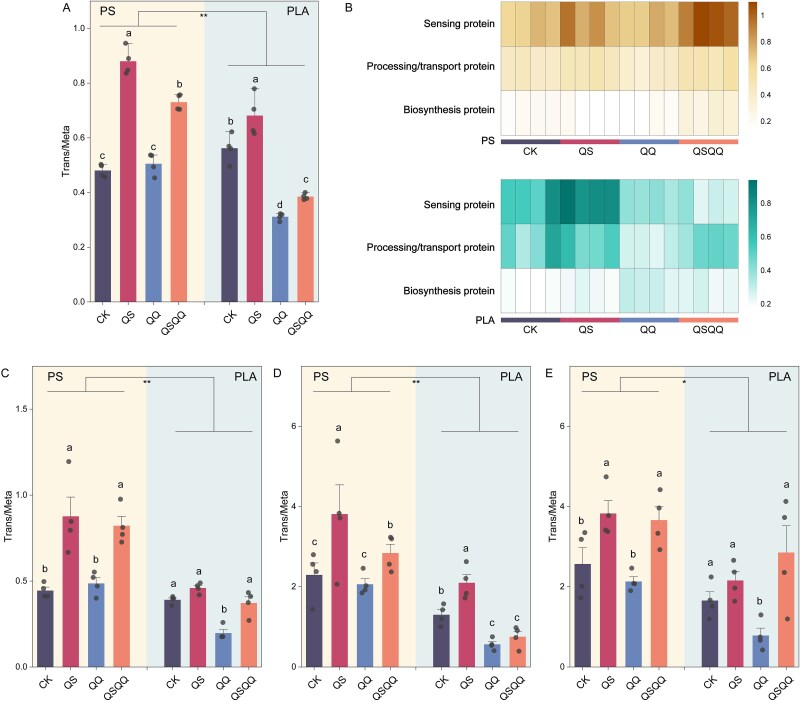
AHL quorum sensing activates transcriptional pathways crucial for biofilm formation and motility. DNA-normalized transcriptional activities (RNA/DNA ratio) of functional pathways quantified from metatranscriptomic data relative to metagenomic potential. (A) DNA-normalized transcriptional activity of overall quorum-sensing pathways in plastispheres under different treatments. (B) DNA-normalized transcriptional activities of three sub-sections within the quorum-sensing pathway across treatments: biosynthesis, processing/transport, and sensing. DNA-normalized transcriptional activities of the overall biofilm formation pathway (C), bacterial chemotaxis (D), and flagellar assembly (E) in plastispheres under different treatments. In (A, C, D, and E), different letters indicate statistically significant differences between treatments (Kruskal–Wallis test with Dunn’s multiple comparisons, *P* < .05). Error bars show the standard deviation of the mean. Asterisks indicate statistically significant differences between PS and PLA plastispheres within the same treatment (^*^P < .05, ^**^P < .01).

To provide an unbiased comparison of gene expression levels relative to potential, we calculated gene-normalized transcript abundances using the ratio of transcript abundances (RNA) to predicted gene abundances (DNA) (RNA/DNA ratio). Consistent with the metatranscriptomic trends, the highest active transcription of overall QS functions was observed in QS treatments, whereas the lowest RNA/DNA values were observed in QQ treatments across both polymer types (Fig. 5A). For instance, in the PS plastisphere, the mean ± standard error of the RNA/DNA values for QS pathways in QS- and QSQQ-treated samples was 0.879 ± 0.043 and 0.731 ± 0.024, respectively, significantly higher than in CK and QQ treatments (0.481 ± 0.020 and 0.505 ± 0.034, respectively, *P* < .05). Further analysis of the complete QS pathway revealed differential regulation of its subsections under varying treatments (Fig. 5B). Although biosynthesis genes were downregulated in QS treatments and upregulated in QQ treatments, genes involved in signal processing/transport and sensing exhibited higher expression in QS treatments and were inhibited in QQ treatments across both PS and PLA plastisphere. This pattern suggests that exogenous AHLs do not necessarily increase biosynthesis gene expression but rather activate the downstream components involved in signal reception and signal transduction, thereby efficiently triggering the QS response.

Both gene abundance and, more critically, expression of the overall biofilm formation pathway were higher in QS- and QSQQ-treated plastispheres compared to CK and QQ treatments (Supplementary Fig. S12). Additionally, the DNA-normalized transcript abundances for the biofilm pathway were also significantly upregulated by QS treatments (Fig. 5C). Expression of critical pathways associated with initial attachment and motility (i.e. bacterial chemotaxis and flagellar assembly) was also enhanced by the AHL molecule and inhibited by AHL acylase. Consistent with these pathway-level observations, we observed elevated gene abundance and transcriptional activity in QS-treated plastispheres for key genes implicated in these processes (Supplementary Figs S13 and S14), including *secA* and *bapA* (associated with protein secretion and surface adhesion) [48, 49], *hfq* (a post-transcriptional regulator influencing the QS response) [50], *flhD* and *flgM* (regulators of flagellar assembly) [51], *gsp* (involved in biofilm matrix production) [52], and *mcp* (a bacterial chemotaxis receptor) [53]. These findings demonstrate at the molecular level how AHLs activate the QS cascade, leading to the transcriptional upregulation of genes necessary for biofilm formation and related processes on microplastic surfaces.

### Acyl-homoserine lactone quorum sensing elevates plastisphere pathogenicity and virulence potential

To assess whether AHL-stimulated QS is associated with shifts toward higher pathogen- and virulence-related functions, we annotated the metagenomic (potential) and metatranscriptomic (activity) data against the PHI database. Both analyses indicated higher relative abundances and transcriptional activities of pathogen-associated functions in the plastisphere samples than in the surrounding water (Supplementary Fig. S15). Within the experimental plastisphere, QS-treated communities showed significantly elevated pathogen-associated signatures compared to CK and QQ treatments (Supplementary Figs S16 and S17). For instance, the metagenomic abundance of human pathogens in QS-treated PS and PLA plastisphere (1069 ± 378 and 1187 ± 108 TPM) was significantly (*P* < .05) higher than in CK and QQ treatments (129–954 TPM). Similar trends indicating elevated pathogenic potential were observed at the transcriptional level. Metagenomic annotation highlighted taxa frequently associated with opportunistic pathogen association (e.g. *Acinetobacter baumannii*, *Pseudomonas aeruginosa*, and *Salmonella enterica*) among the most abundant pathogen-associated organisms (Supplementary Fig. S16D), whereas metatranscriptomic annotation indicated active expression from both bacterial and fungal pathogen-associated groups (e.g. *Burkholderia glumae, Cryptococcus neoformans*, and *Fusarium graminearum*) (Supplementary Fig. S17D). Together, these analyses suggest an elevated virulence potential in QS-promoted plastispheres, even though noting that database annotations reflect potential rather than direct infection phenotypes.

**Figure 6:**
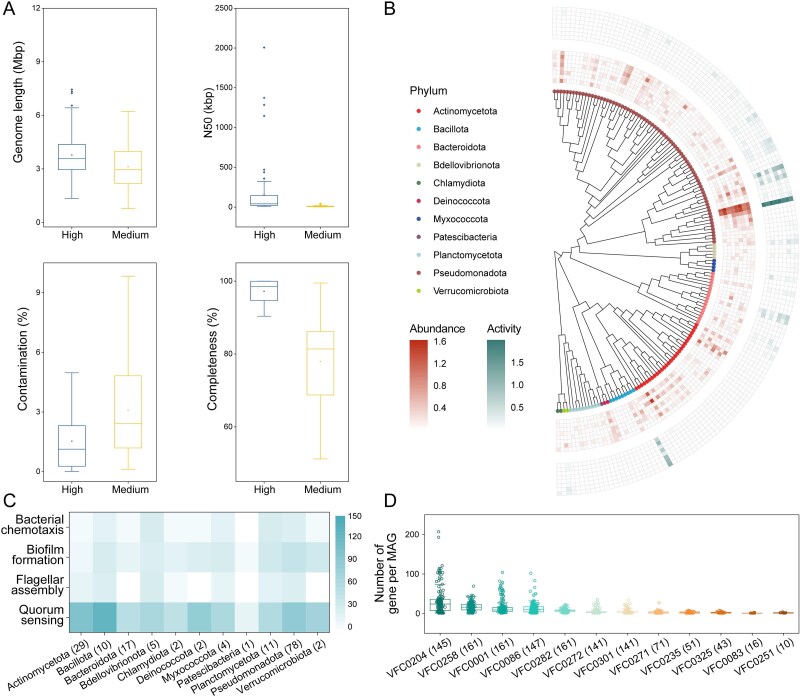
Genomic potential and activity of MAGs from experimental plastispheres reveal virulence factor repertoire. (A) Box plots showing key characteristics of metagenome-assembled genomes (MAGs) obtained from experimental plastisphere samples, including N50, genome length, completeness, and contamination, separated by MAG quality (medium vs. high quality). (B) Maximum likelihood phylogenetic tree of the MAGs. Gradient color from white to red represents average MAG abundance, and gradient color from white to green represents average MAG transcriptional activity across treatments. (C) Occurrence (presence/absence) of genes involved in core functional categories related to quorum sensing, biofilm formation, chemotaxis, and flagellar assembly across the MAGs. (D) Occurrence (presence/absence) of genes involved in different categories of virulence factors (VFs) across the MAGs, annotated against the Virulence Factor Database (VFDB). VFC0204, Motility; VFC0258, Immune modulation; VFC0301, Regulation; VFC0086, Effector delivery system; VFC0282, Stress survival; VFC0272, Nutritional/Metabolic factor; VFC0001, Adherence; VFC0271, Biofilm; VFC0251, Exoenzyme; VFC0235, Exotoxin; VFC0083, Invasion, VFC0325, Antimicrobial activity/Competitive advantage. The numbers in parentheses correspond to the number of MAGs.

To investigate the genomic basis of this virulence potential, particularly in taxa enriched by AHLs, we performed a focused analysis of 161 MAGs derived from the experimental data, including 85 high-quality genomes (completeness >90% and contamination <5%) (Fig. 6A). These MAGs represented diverse taxa, with notable representation from Pseudomonadota (78 MAGs) and Actinomycetota (29 MAGs), and collectively harbored key functional genes related to QS, biofilm formation, chemotaxis, and flagellar assembly across multiple phyla (Fig. 6B and C). Highlighting a specific genome among these, MAG12, identified as the potential pathogen *A. lwoffii*, exhibited particularly high abundance and transcriptional activity within the QS-treated plastisphere, providing a genomic link between QS-driven selection and virulence-associated potential. Analysis against the Virulence Factor Database (VFDB) identified a total of 15 267 potential VFs across these MAGs (Fig. 6D). Among these, VFs involved in immune modulation (2742), adherence (2629), and stress survival (1167) were consistently present in all MAGs. VFs related to motility (4640), effector delivery system (2070), nutritional/metabolic factor (745), regulation (638), and biofilm (218) were identified in over half of the MAGs. Furthermore, analysis against the structured database of ARGs (SARG) revealed that all recovered MAGs contain at least one ARG (Supplementary Fig. S18), with MAG 77 harboring the maximum number of ARGs (55 ARGs). The total ARG abundance varied greatly among different phylogenetic groups, with Gammaproteobacteria (16.4 ± 10.9 ARG counts), Bacilli (14.0 ± 4.6), Actinomycetia (12.8 ± 4.7), Alphaproteobacteria (8.0 ± 5.1), and Bacteroidia (5.2 ± 2.2) ranking among the top carriers. Collectively, these MAG-based results indicated that taxa enriched under QS-promoting conditions can carry broad repertoires of putative VFs and ARGs, consistent with an elevated virulence potential of the QS-treated plastisphere community.

**Figure 7:**
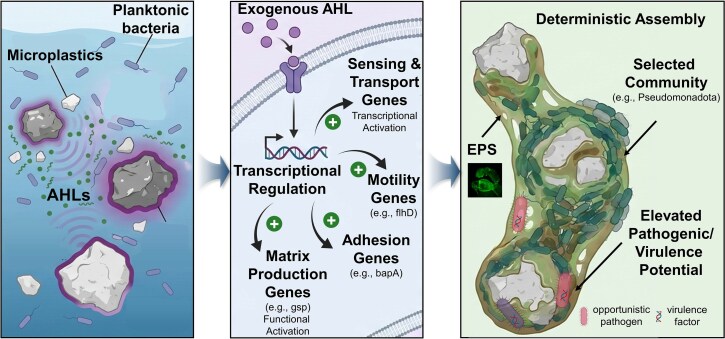
Conceptual paradigm summarizing that AHL quorum sensing can drive plastisphere formation and shift risk-relevant functions. AHL signals can accumulate at the plastic–water interface, activate QS sensing/transport, and promote early attachment and maturation processes (flagellar motility, surface adhesion, and EPS/matrix production). These QS-driven traits increase biofilm biomass and restructure community assembly toward deterministic selection, enriching QS-responsive taxa and reshaping microbial interaction networks. Coregulation of biofilm and virulence-associated functions may elevate virulence potential within QS-promoted plastispheres, including enrichment and activation of pathogen and virulence-related signatures.

## Discussion

The plastisphere represents a critical ecological habitat emerging in the Anthropocene, harboring distinct microbial communities with unique functional profiles. Our integrated global meta-analysis and controlled experiments identify AHL-mediated QS as a key driver of plastisphere biofilm assembly. Across public datasets, plastisphere communities consistently showed enrichment of QS and biofilm formation genes, with a pronounced signal for AHL QS. A significant correlation between these functional gene categories was observed. Even though correlation alone cannot establish causation, this finding provided compelling initial support for the hypothesis that QS plays a key role in plastisphere establishment. By directly manipulating an AHL signal and a QQ enzyme under continuous flow conditions, we then demonstrated causal links between QS activity, EPS production, 3D biofilm architecture, and community assembly outcomes. Together, these results support a mechanistic view in which chemical communication helps coordinate attachment, motility, and matrix production to accelerate plastisphere formation (Fig. 7).

At the molecular level, our integrated metagenomic and metatranscriptomic data elucidated how the AHL signal promotes plastisphere formation. We found that AHL treatment specifically activated the transcription of genes encoding surface protein, extracellular polysaccharides, bacterial chemotaxis receptors (*mcp*), and flagellar assembly components (*flhD*, *flgM*). These functional categories are crucial for the initial stages of biofilm development, including motility toward the substrate (chemotaxis, flagella) [54, 55], initial reversible attachment (surface proteins, flagella) [55], and the subsequent irreversible attachment and biofilm matrix formation (EPS, surface proteins). This transcriptional reprogramming demonstrates that AHL-induced quorum sensing promotes the adhesion of bacterial cells to the plastic surface and cohesion among cells, thereby directly driving plastisphere development. Our sequencing analyses revealed a nuanced regulatory pattern: exogenous AHL addition upregulated genes involved in signal sensing and processing/transport while simultaneously downregulating endogenous AHL biosynthesis genes. This pattern is consistent with negative feedback regulation observed in many QS circuits, where an external supply of autoinducers effectively triggers the quorum threshold, reducing the metabolic burden to synthesize new signals while still potently activating receptor-mediated transcriptional programs downstream. These results highlight that, in multispecies plastispheres, QS manipulation can decouple signal production potential from QS activity, emphasizing the critical value of RNA/DNA-based activity metrics for interpreting QS function.

Beyond driving biofilm formation, exogenous AHL stimulation exerted a strong selection pressure on plastisphere community structure, leading to the lowest richness and Shannon Index. Because biofilm biomass and EPS increased rather than decreased under QS treatment, the reduced diversity is unlikely to be explained solely by overt toxicity; instead, it is consistent with competitive dominance by AHL-responsive, biofilm-adapted taxa under high-density conditions (e.g. the enrichment of Proteobacteria under QS treatments) [56]. Consistent with our findings, an isolation and culture survey of marine microplastic biofilm also revealed that culturable bacteria associated with these surfaces are frequently characterized with QS systems and significant biofilm-forming abilities [57]. The clustering pattern of communities (QS/QSQQ separated from CK/QQ and planktonic) directly reflects this AHL-driven selective recruitment of biofilm-forming microbes.

Co-occurrence network analysis revealed less complex interaction and a higher proportion of inferred competitive relationships (negative edges) within the denser, QS-enhanced plastispheres compared to controls. This increased competition likely arises from resource limitation under high cell density, with QS enabling coordinated behaviors for competitive advantage [58]. In ecological theory, competitive interactions can stabilize population dynamics by constraining unchecked growth [59, 60], but network stability in this sense is not equivalent to robustness to perturbation. Our node removal analyses indicated that these QS/QSQQ networks were less robust, which can be reconciled by considering the macro-architecture of the community. The strong selection exerted by exogenous AHLs drastically reduced overall network complexity, size, and diversity. Consequently, although the specialized taxa within the QS-driven plastisphere engage in intense competition to maintain their specific niches, the community as a whole lacks the structural redundancy provided by a larger, more diverse microbiome. The AHL molecule promotes the formation of a highly specialized, fiercely competitive plastisphere biofilm.

Across both imaging and multi-omics datasets, we detected no significant differences between PS and PLA plastisphere within the same treatment for the key metrics. This suggests that, under our conditions, QS manipulation exerts a stronger control over plastisphere formation than the PS versus PLA polymer contrast. Although polymer-specific effects on biofilm assembly are typically governed by surface physiochemistry (e.g. roughness, hydrophobicity), additive chemistry, and environmental aging, both PS and PLA possess hydrophobic surfaces. This shared hydrophobicity may similarly facilitate the adsorption and local concentration of lipophilic AHL molecules, creating comparable “QS hotspots” on both polymers. Furthermore, similar surface roughness between PS and PLA after incubation may have contributed to this polymer-independence observation (Supplementary Fig. S20). To fully disentangle these dynamics, future tests across additional commodity plastics (e.g. PE, PP, PVC, PBAT) and environmentally weathered particles to quantify how polymer traits modulate QS-driven colonization.

Microplastics are increasingly recognized as potential reservoirs and vectors for pathogens in the environment [12, 61]. Our PHI/VFDB-based annotations and MAG analyses suggest that QS-promoted plastisphere can exhibit elevated virulence potential, including enrichment of opportunistic pathogen-associated taxa (e.g. Acinetobacter) and increased expression of virulence-related function. This aligns with the known phenomenon that QS systems often coregulate both biofilm formation and VFs, as these traits share common regulatory elements or functional dependencies. This observation is consistent with well-established coregulation of biofilm and virulence traits in many pathogens (e.g. *Erwinia carotovora*, *Staphylococcus aureus*, *P. aeruginosa,* and *Vibrio cholerae*) via QS [62–64]. Even though our results only reflect genomic and transcriptomic signatures rather than demonstrated infection outcomes, these findings provide a mechanistic hypothesis linking plastisphere biofilm development to risk relevant functional shifts.

Our study suggested that interfering with AHL-mediated QS may represent a promising strategy to mitigate the pathogenic risks associated with plastispheres. A previous study has shown that the degradation intermediate of the polymer polyhydroxy butyrates (PHBs) could effectively inhibit biofilm formation and AHL-mediated QS pathway in the *Vibrio* PUGSK8 [65]. Additionally, the application of anti-quorum-sensing compounds coatings on material surface has also demonstrated potential for controlling bacterial pathogenicity and biofilm dynamics [66]. Deploying quorum quenching agents or designing next-generation plastics with surfaces engineered to inhibit QS signaling should be considered in the design of future plastic materials. However, we strongly caution that in a complex, open environment, broad QS quenching could have unintended effects on beneficial microbial functions, such as nutrient cycling. Any applied strategy must be highly targeted and paired with ecological risk assessment to evaluate off-target impacts and evolutionary responses.

Although this study establishes a robust mechanistic foundation for QS-driven plastisphere assembly, its broader ecological relevance and inherent limitations should be considered. Our meta-analysis evaluated a global snapshot encompassing diverse marine and freshwater plastispheres, whereas our experimental validation used wastewater effluent. This nutrient-rich environment was chosen because wastewater treatment plants are major conduits for microplastics entering aquatic ecosystems, representing a critical stage for initial biofilm seeding. However, wastewater-derived communities are nutrient-rich and may contain elevated densities of QS-capable bacteria, potentially amplifying QS effects compared with oligotrophic seawater. Our findings mechanistically validate an AHL-QS driver that is broadly encoded, though its ecological magnitude will inherently vary by ecosystem and community context. Future work should extend this framework to inocula from marine and freshwater environments under more representative physicochemical regimes. We utilized 10 μg/l (≈ 50 nM) of 3OC6-HSL, which is within the range commonly used to elicit QS-dependent biofilm responses and aligns with the experimentally determined QS-response thresholds for initial adhesion of wastewater biofilm [34]. Although this dose represents a plausible high-local-exposure scenario designed to test mechanistic potential, future dose–response experiments and *in situ* AHL measurements on plastic-associated biofilm will be essential to constrain real-world relevance across diverse ecosystems. We cannot fully exclude non-QS effects of exogenous AHL addition (e.g. metabolic utilization of signal molecules). Future studies should incorporate comprehensive dose–response experiments paired with *in situ* chemical measurements. Finally, although AHL signals dominated our meta-analysis, other QS systems (e.g. AI-2, AIP, and c-di-GMP) were also detected and may contribute to plastisphere development through cross-talk or taxa-specific regulation. Deciphering how multiple signaling systems interact on plastics remains an important frontier for plastisphere ecology.

Our comprehensive investigation provides compelling evidence for the significant role of AHL-mediated QS in shaping plastisphere characteristics. We have demonstrated that the AHL molecule actively promotes biofilm formation on both conventional and biodegradable microplastics by transcriptionally upregulating key genes involved in surface adhesion, matrix production, and motility. We have uncovered a concerning link between AHL-mediated QS and the expression of potential pathogens within the plastisphere, highlighting the role of this signaling mechanism in potentially elevating the risks associated with plastic pollution. Given the ubiquity of plastic particles and their associated plastisphere, they may introduce pathogens and other microorganisms into diverse environments, posing an invasion risk to natural ecosystems and increasing the uncertainty of infection. Recognizing this, our study suggests that disrupting this communication system could be a promising avenue for mitigating the global risks associated with plastic pollution, and we propose that future research should focus on manipulating bacterial signaling systems as a means to reduce these emerging ecological and health risks. These findings advance our understanding of the biological dimensions of plastic pollution in Anthropocene ecosystems.

## Supplementary Material

SI_r2_wrag066

## Data Availability

Raw amplicon and metagenomic sequencing data are deposited in the National Genomics Data Center (https://ngdc.cncb.ac.cn/) under the accession number PRJCA055412. Code for the figures is available at https://github.com/watertimes/AHL_and_plastisphere.
