# Supplementary material for: AHL-mediated quorum sensing drives plastisphere formation and elevates pathogenic potential
Source: ISME J. 2026 Mar 24;20(1):wrag066. doi: 10.1093/ismejo/wrag066 (PMC13122622; doi:10.1093/ismejo/wrag066)
Supplement: SI_r2_wrag066 [file si_r2_wrag066.docx]

**Supporting Information for:**

**AHL-mediated quorum sensing drives plastisphere formation and elevates pathogenic potential**

Jie Wang ^1,*^, Lijia Lu ^1^, Yuanze Sun ^2^, Lauren F. Messer ^3^, Mochen Wu ^1^, Zhuoran Duan ^1^, Jia Shi ^1^, Yuyi Yang ^4^, Changchao Li ^5^, Yanping Mao ^6^, Dong Zhu ^7^, Matthias C. Rillig ^2^, Xiaoping Wang ^1,*^

^1^ China Agricultural University, Beijing 100193, China

^2^ Institute of Biology, Freie Universität Berlin, Altensteinstrasse 6, 14195 Berlin, Germany

^3^ Organisms and Ecosystems, Earlham Institute, Norwich, NR4 7UZ, UK

^4^ Research Center for Environmental Ecology and Engineering, School of Environmental Ecology and Biological Engineering, Wuhan Institute of Technology, Wuhan 430205, PR China

^5^ Department of Civil and Environmental Engineering, The Hong Kong Polytechnic University, Hung Hom, Kowloon, Hong Kong 999077, China

^6^ College of Chemistry and Environmental Engineering, Shenzhen University, Shenzhen, Guangdong 518071, China

^7^ Key Laboratory of Urban Environment and Health, Ningbo Urban Environment Observation and Research Station, Institute of Urban Environment, Chinese Academy of Sciences, Xiamen 361021, Peoples Republic of China

* Corresponding author:

Xiaoping Wang

Email: [wangxp@cau.edu.cn](mailto:wangxp@cau.edu.cn)

ORCID: 0000-0001-7524-7540

Jie Wang

Email: [jiewangcau@cau.edu.cn](mailto:jiewangcau@cau.edu.cn)

ORCID: 0000-0001-5657-8109

**Amplicon sequencing data analysis**

The alpha diversity, including Chao1 richness and Shannon Index, was estimated using “Vegan” package in R. Kruskal-Wallis test with Dunn’s multiple comparisons was used to determine significant differences between different treatments, and a P value of < 0.05 was regarded as statistically significant. Principal-coordinates analysis (PCoA) based on Bray-Curtis dissimilarity was used to visualize the beta diversity of different samples, and permutational multivariate analysis of variance (PERMANOVA) was used to test the significance. To explore differences in the co-occurrence interactions among species in different plastispheres, the network at the ZOTU level was carried out via the Molecular Ecology Network Analysis pipeline (MENA) ((<http://ieg4.rccc.ou.edu/mena>). All networks were constructed on the basis of Pearson correlations of ASV abundances, followed by a random matrix theory-based approach that determines the correlation cut-off threshold in an automatic fashion. The same cutoff value (0.91) was used in the current study. To ensure the reliability of correlation calculation, only ZOTUs present in all six replicates were included for calculation. The topological indexes of the MENA networks, including the node, the link, the average degree, the clustering coefficient, the average geodesic distance, and the modularity, were estimated. Random networks were generated for each network by randomly rewriting the links among the nodes while constraining node and edge numbers, following the Maslov-Sneppen procedure. The topological roles of different nodes in the microbial networks were classified four categories based on inter-module connectivity (Pi) and intra-module connectivity (Zi): (1) peripheral nodes (Zi ≤ 2.5, Pi ≤ 0.62); (2) connectors (Zi ≤ 2.5, Pi > 0.62); (3) module hubs (Zi > 2.5, Pi > 0.62); and (4) network hubs (Zi > 2.5, Pi > 0.62). Nodes belonged to connectors, module hubs, and network hubs were identified as the keystone taxa. We further compared the network stability of different plastispheres via the robustness metric, which describes the proportion of the remaining species in the network after random node removal, as well as, cohesion, an abundance-weighted, null model-corrected metric based on pairwise correlations across taxa to reflect the degree of cooperative behaviors or competitive interactions.

A modified framework to quantitatively infer community assembly mechanisms by phylogenetic bin-based null model analysis (iCAMP) was used to estimate the potential contribution of different types of community assembly processes. The process is identified based on null model analysis of the phylogenetic diversity using beta Net Relatedness Index (βNRI), and taxonomic β-diversities using modified Raup–Crick metric (RC). The fraction of pairwise comparisons with βNRI < −1.96 is considered as the percentages of homogeneous selection, whereas those with βNRI > +1.96 as the percentages of heterogeneous selection. Taxonomic diversity metric RC is used to partition the remaining pairwise comparisons with |βNRI| ≤ 1.96. The fraction of pairwise comparisons with RC < −0.95 is treated as the percentages of homogenizing dispersal, whereas those with RC > + 0.95 as dispersal limitation. The remains with |βNRI| ≤ 1.96 and |RC| ≤ 0.95 represent the percentages of drift. Normalized stochasticity ratios (NST) based on phylogenetic metrics (pNST) was also estimated to quantify the relative importance of deterministic and stochastic processes with 50% as the boundary point between more deterministic (< 50%) and more stochastic (> 50%).

Table S1. The metagenomic data used in this study

| Article | Location | Polymer | Ecosystem | Reference | Source |
| --- | --- | --- | --- | --- | --- |
| Shotgun Metagenomics Reveals the Benthic Microbial Community Response to Plastic and Bioplastic in a Coastal Marine Environment | The Laguna Madre (Texas, USA) | PET, PHA, seawater | Marine | Pinnell and Turner, Frontiers in Microbiology 2019, 10, 1252 | PRJEB15404 |
| Deciphering the mechanisms shaping the plastisphere antibiotic resistome on riverine microplastics | Huangpu River, China | Field collected MPs and water | Freshwater | Xu et al., Water Research 2022, 225, 119192 | PRJCA011465 |
| Lake plastisphere as a new biotope in the Anthropocene: Potential pathogen colonization and distinct microbial functionality | Taihu Lake, China | Field collected MPs and water | Freshwater | Xu et al., Journal of Hazardous Materials 2024, 461, 132693 | PRJCA011465 |
| Microbial decomposition of biodegradable plastics on the deep-sea floor | Pacific Ocean | P3HB4HB, PBAT, PBSA, PCL, PE, PET, PHA, PHBH, PHBV, PLLA, PP, PS, seawater | Marine | Omura et al., Nature Communications 2024, 15, 568 | PRJNA886482 |
| Plastics select for distinct early colonizing microbial populations with reproducible traits across environmental gradients | West Palm Beach, Florida, US | PP, PE, PMMA, PS, PHA, seawater | Marine | Bos et al., Environmental Microbiology 2023, 25, 2761-2775 | PRJNA777294 |
| Microbial hitchhikers harbouring antimicrobial-resistance genes in the riverine plastisphere | River Sowe, Stoneleigh, UK | LDPE, PP, water | Freshwater | Zadjelovic et al., Microbiome 2023, 11, 225 | PRJEB52400 |
| Ecology and risks of the global plastisphere as a newly expanding microbial habitat | Shandong, China | Field-collected MPs | Freshwater and seawater | Li et al., The Innovation 2024, 5, 100543 | PRJNA984432 |
| Exploring the Composition and Functions of Plastic Microbiome Using Whole-Genome Sequencing | Lake Macquarie, New South Wales, Australia | PP, PS, PVC, PCL, water | Freshwater | Bhagwat et al., Environmental Science & Technology 2021, 55, 4899-4913 | PRJEB41132 |
| Exploring the dynamics of antibiotic resistome on plastic debris traveling from the river to the sea along a representative estuary based on field sequential transfer incubations | Haihe Estuary, China | PET | Freshwater and Marine | Zhao et al., Science of The Total Environment 2024, 923, 171464 | PRJNA1000058 |

Table S2. The topological parameters of the networks in different treatments.

| Topological parameter | PS | | | |
| --- | --- | --- | --- | --- |
|  | CK | QS | QQ | QSQQ |
| node  edge  negative proportion  positive proportion  average degree  average clustering coefficient  average path distance  connectedness  geodesic efficiency  transitivity  modularity  relative | 165  455  0.310  0.690  5.52  0.102  6.11  0.721  0.24  0.364  0.554 | 76  131  0.458  0.542  3.45  0.163  5.33  0.462  0.304  0.426  0.603 | 175  612  0.224  0.776  6.99  0.102  3.771  0.514  0.352  0.414  0.431 | 110  162  0.549  0.451  2.94  0.161  5.627  0.511  0.26  0.178  0.730 |
|  | PLA | | | |
|  | CK | QS | QQ | QSQQ |
| node  edge  negative proportion  positive proportion  average degree  average clustering coefficient  average path distance  connectedness  geodesic efficiency  transitivity  modularity | 128  270  0.482  0.518  4.22  0.296  6.524  0.718  0.228  0.316  0.732 | 83  112  0.545  0.455  2.69  0.081  5.484  0.415  0.284  0.079  0.802 | 130  332  0.474  0.526  5.11  0.212  4.531  0.574  0.313  0.329  0.595 | 108  150  0.513  0.487  2.78  0.192  4.619  0.217  0.353  0.232  0.809 |


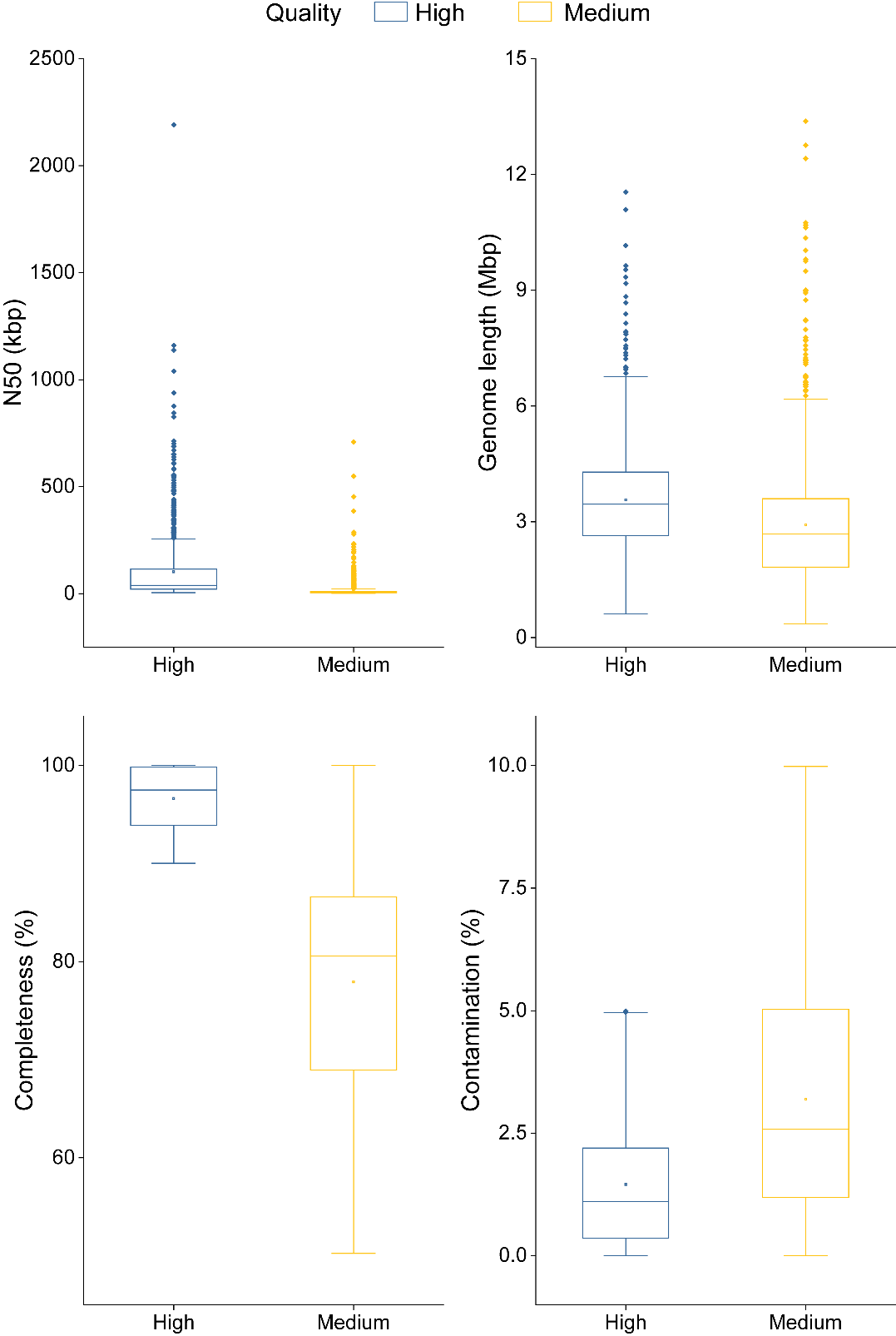


Figure S1. Boxplot display of different characteristics of MAGs (N50 (kbp), genome length (Mbp), completeness (%), and contamination (%)). The MAGs are separated on the basis of their quality (medium or high).


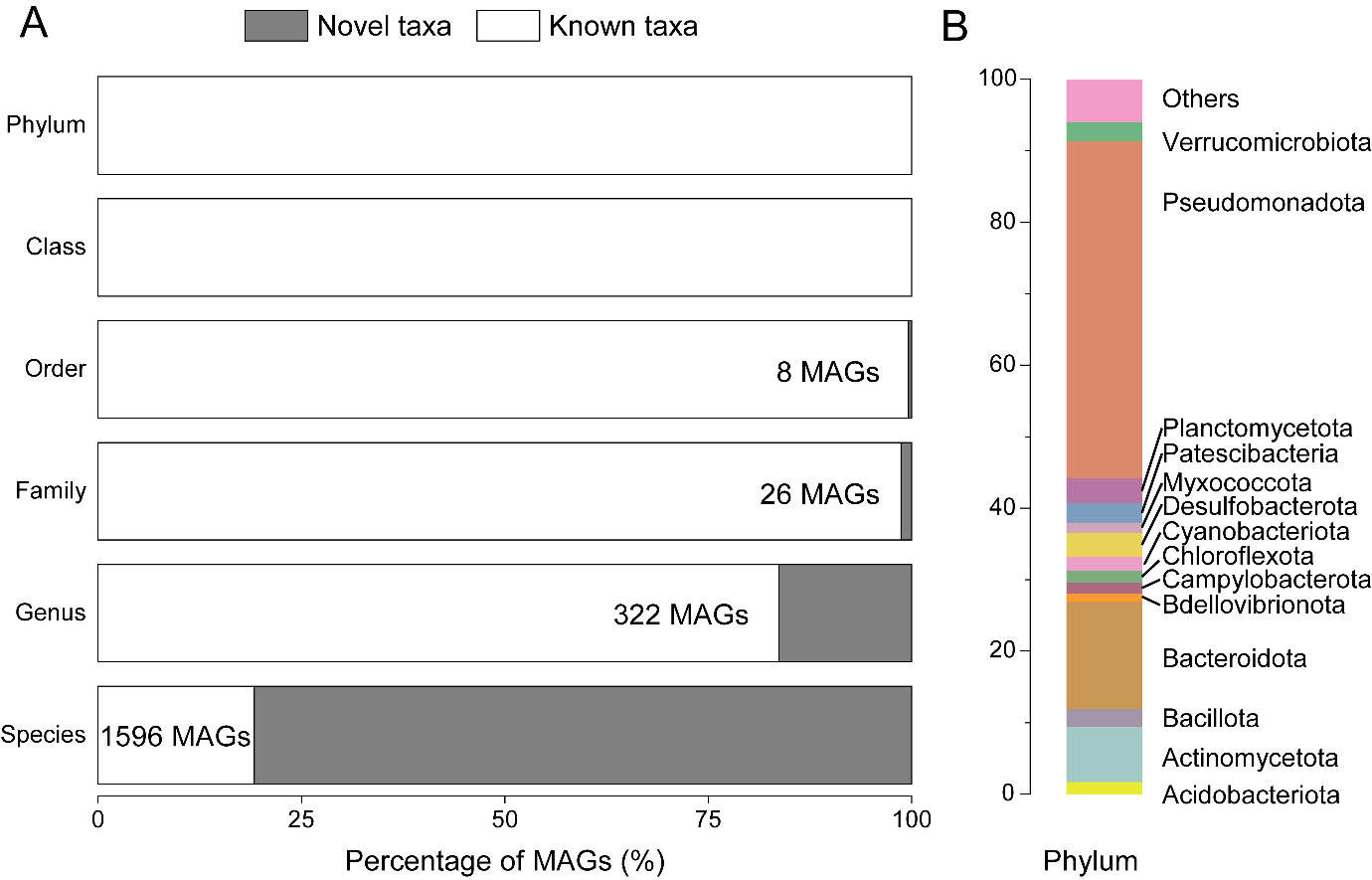


Figure S2. Information of metagenome-assembled genome (MAGs). A. Stacked bar plot for novelty quantification of the MAGs at different taxonomic levels. B. The taxonomic affiliation of the MAGs at the phylum level.


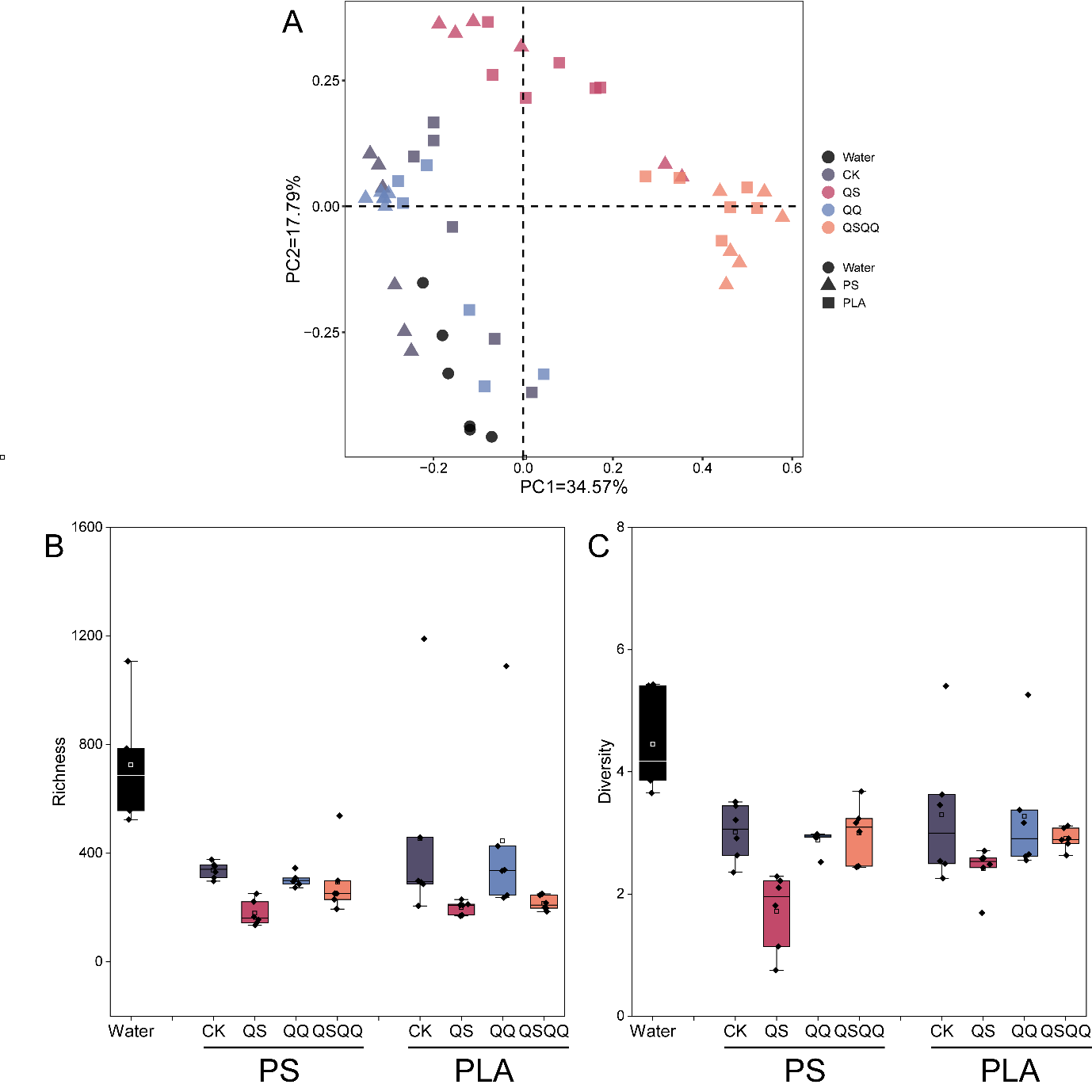


Figure S3. Alpha and beta diversity of tested communities. A. Principal coordinates analysis showing the community differences between water and plastisphere samples in the incubated experiment. The Chao1 richness B and Shannon Index C.


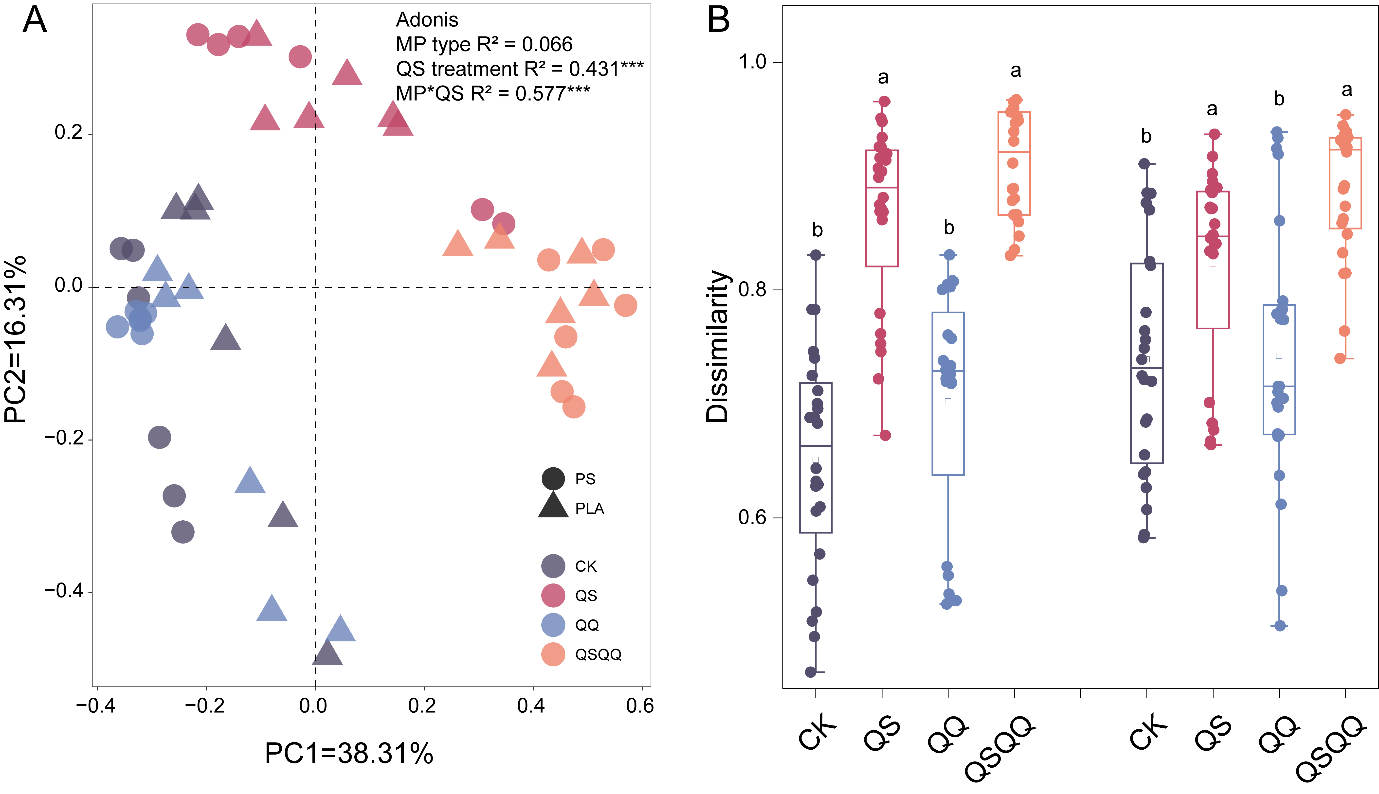


Figure S4. Differences among the plastisphere. A. Principal coordinates analysis showing the difference among the plastisphere samples under different QS treatments. B. The dissimilarity of plastisphere and water samples among different treatments.


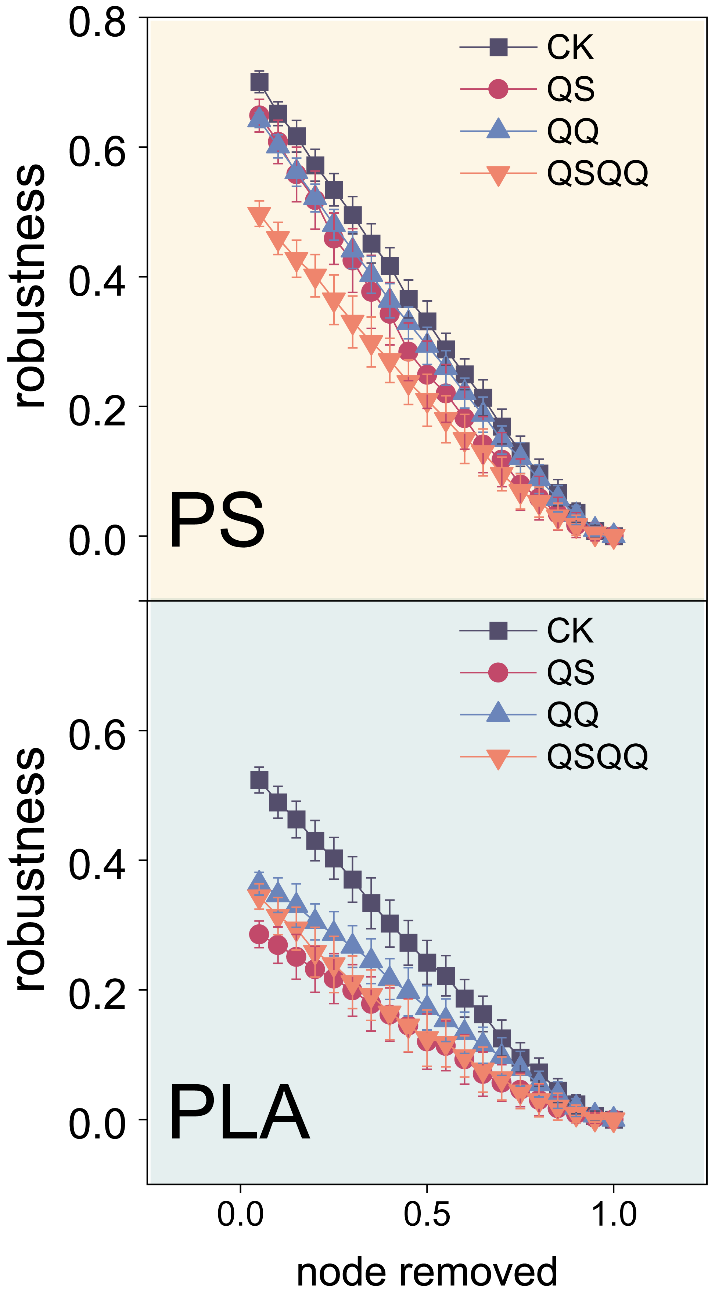


Figure S5. The robustness of different networks.


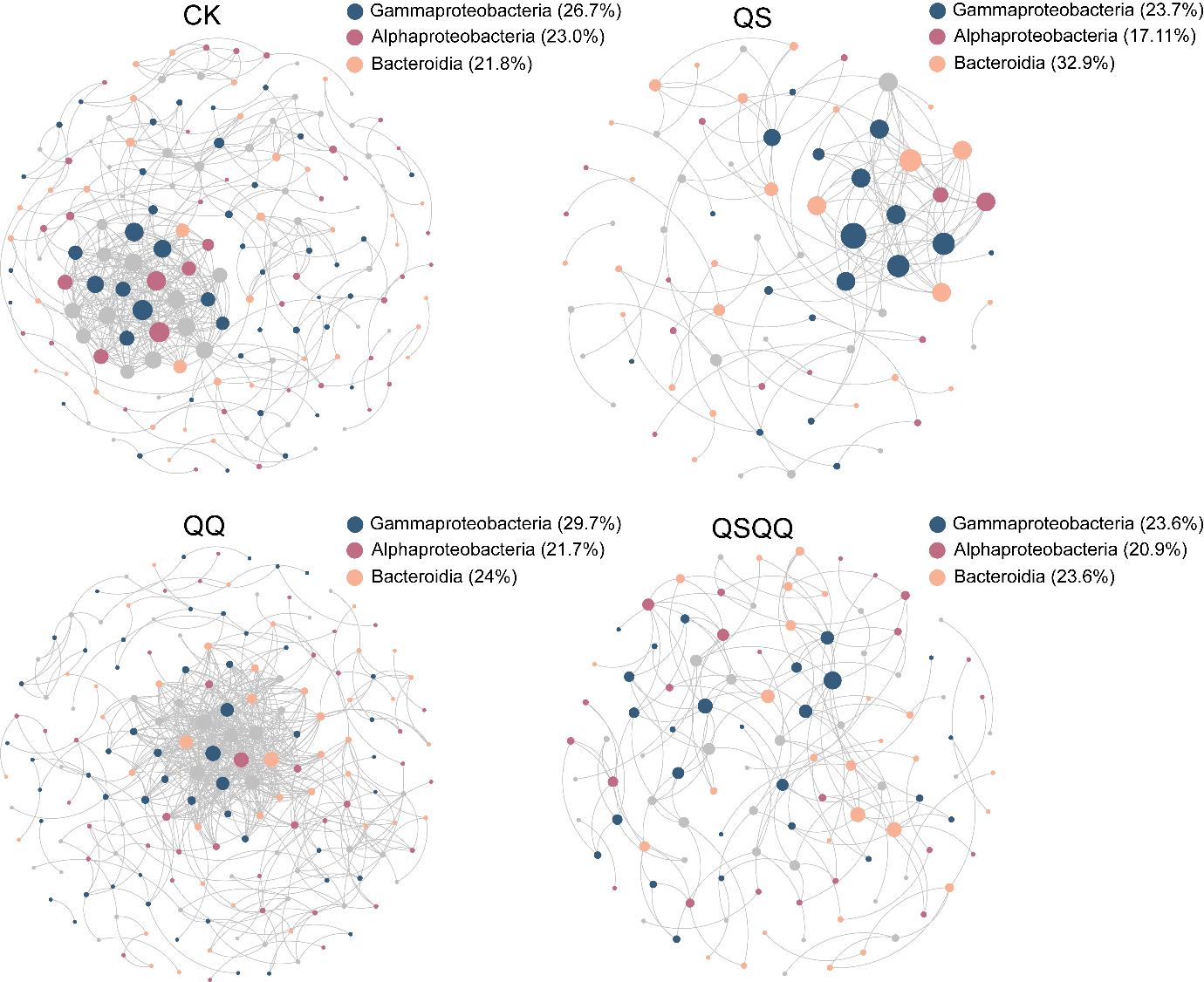


Figure S6. The networks and dominant nodes in PS plastisphere under different treatments.


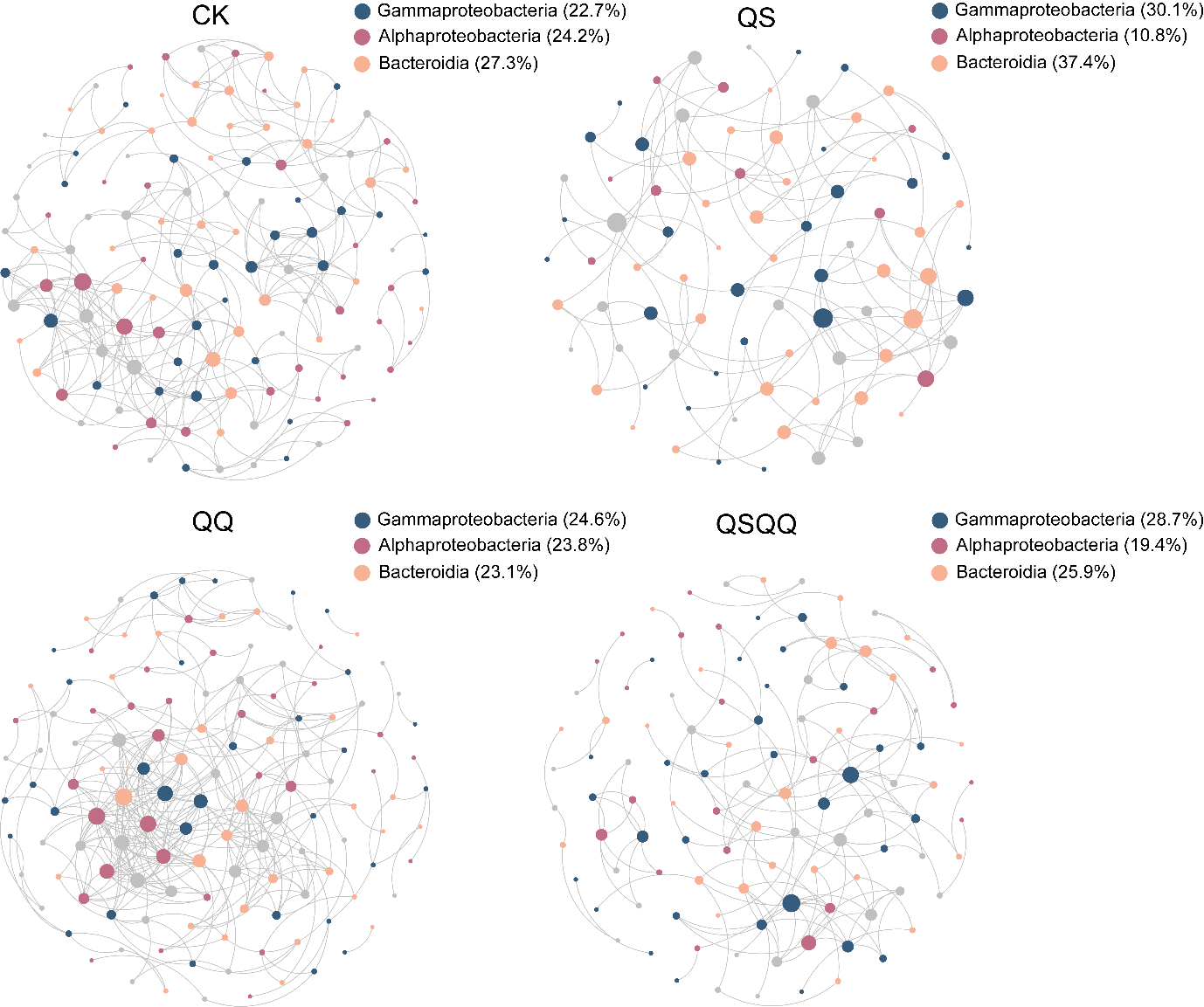


Figure S7. The networks and dominant nodes in PLA plastisphere under different treatments.


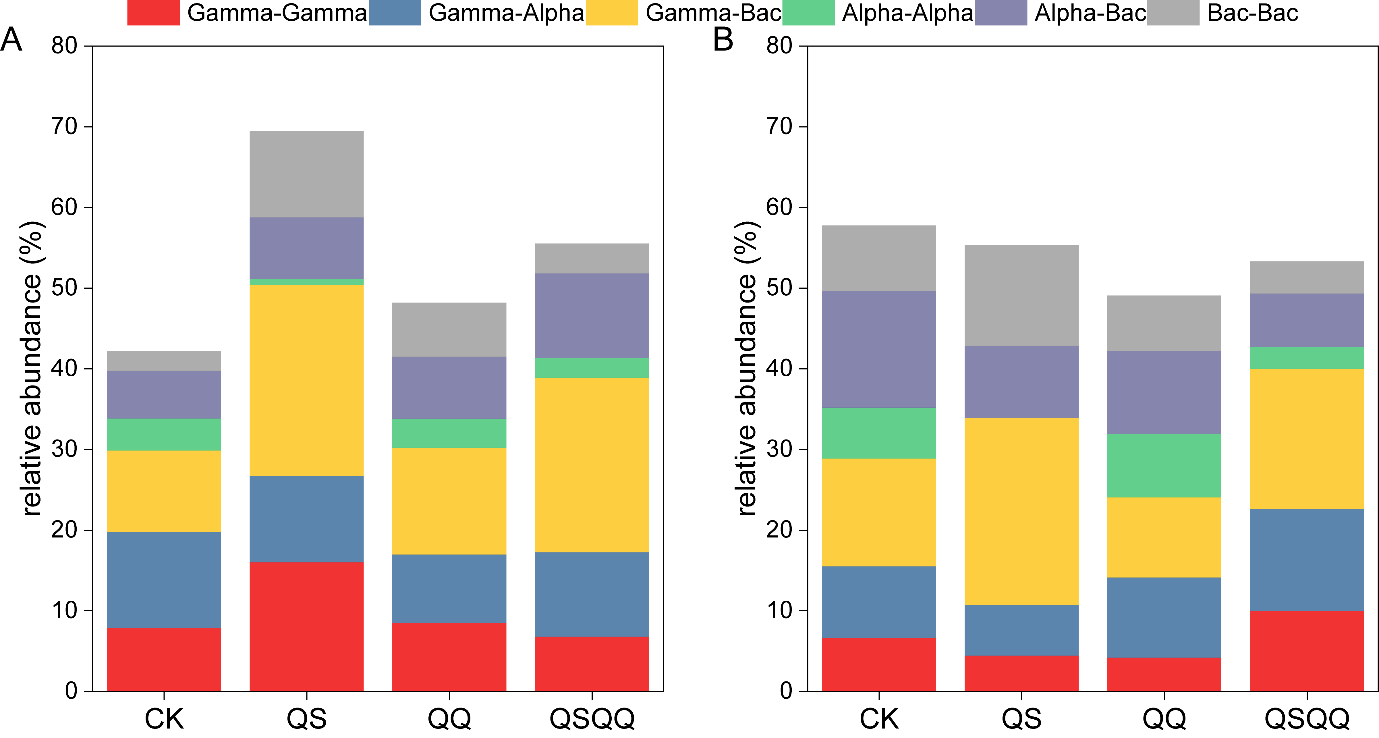


Figure S8. The proportion of dominant edges in each network. A. PS networks; B. PLA networks.


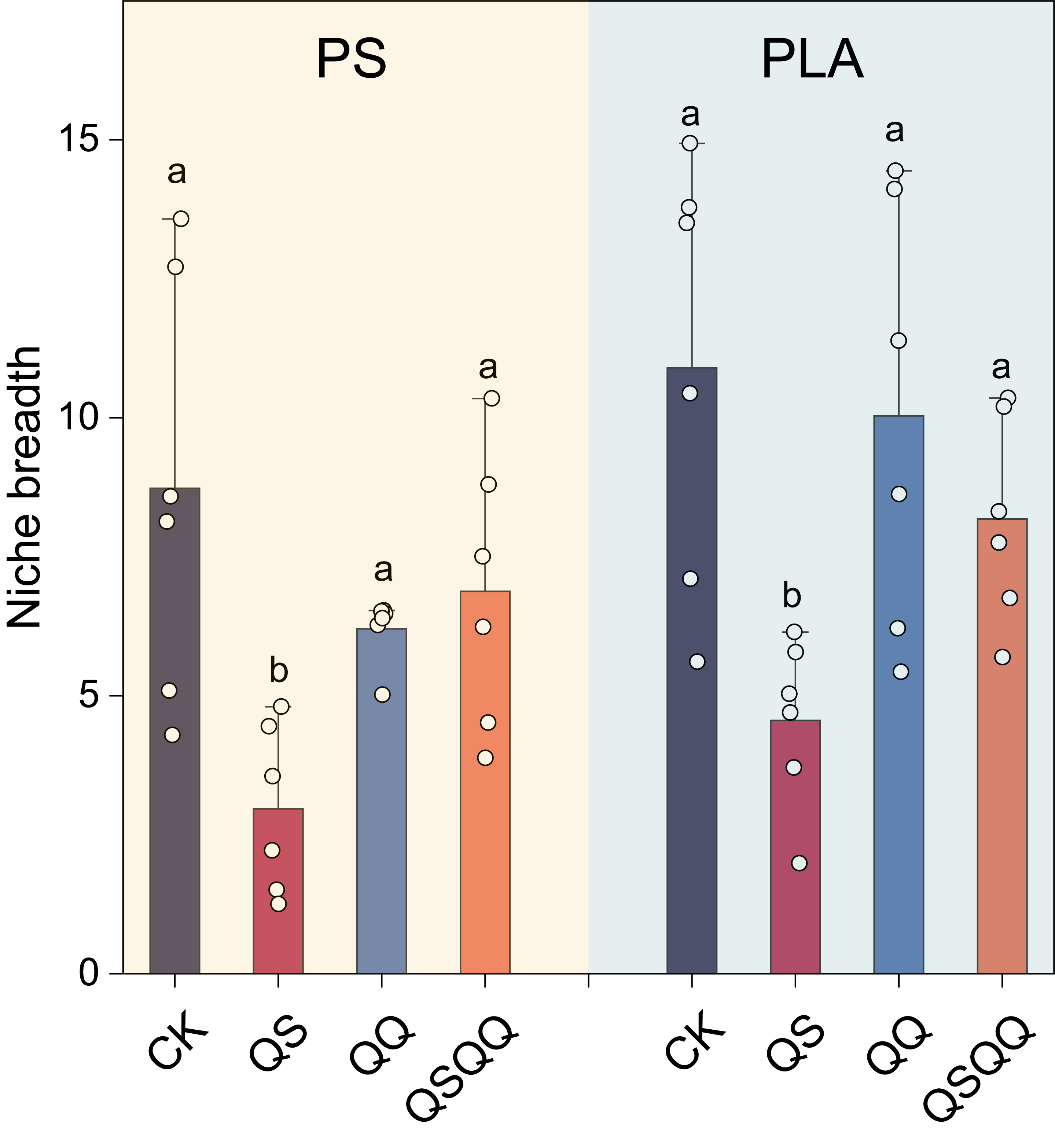


Figure S9. The niche breadth in each treatment.


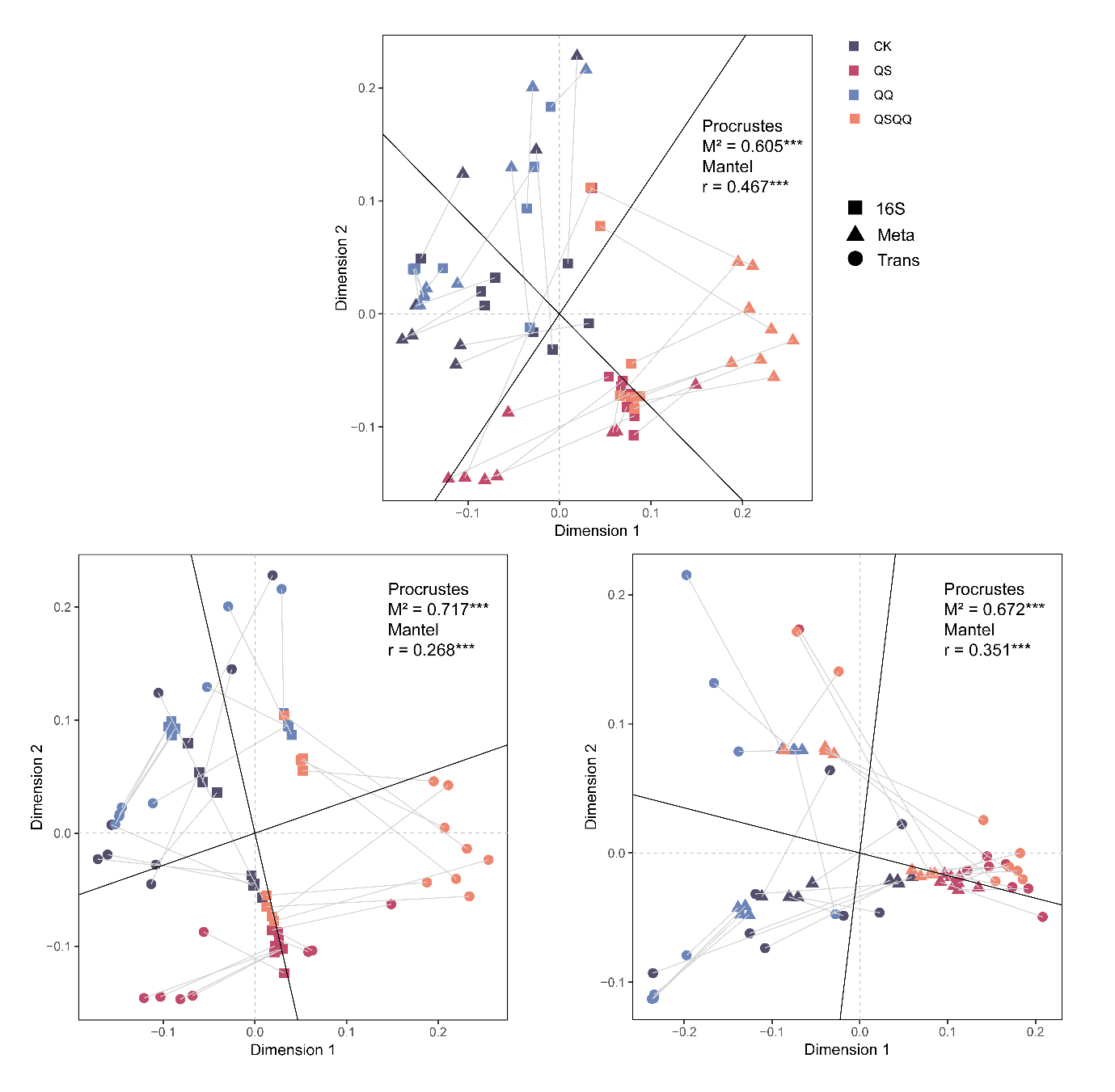


Figure S10. The Procrustes analysis and mantel test among plastisphere community structure, predicted functional potential, and active gene expression.


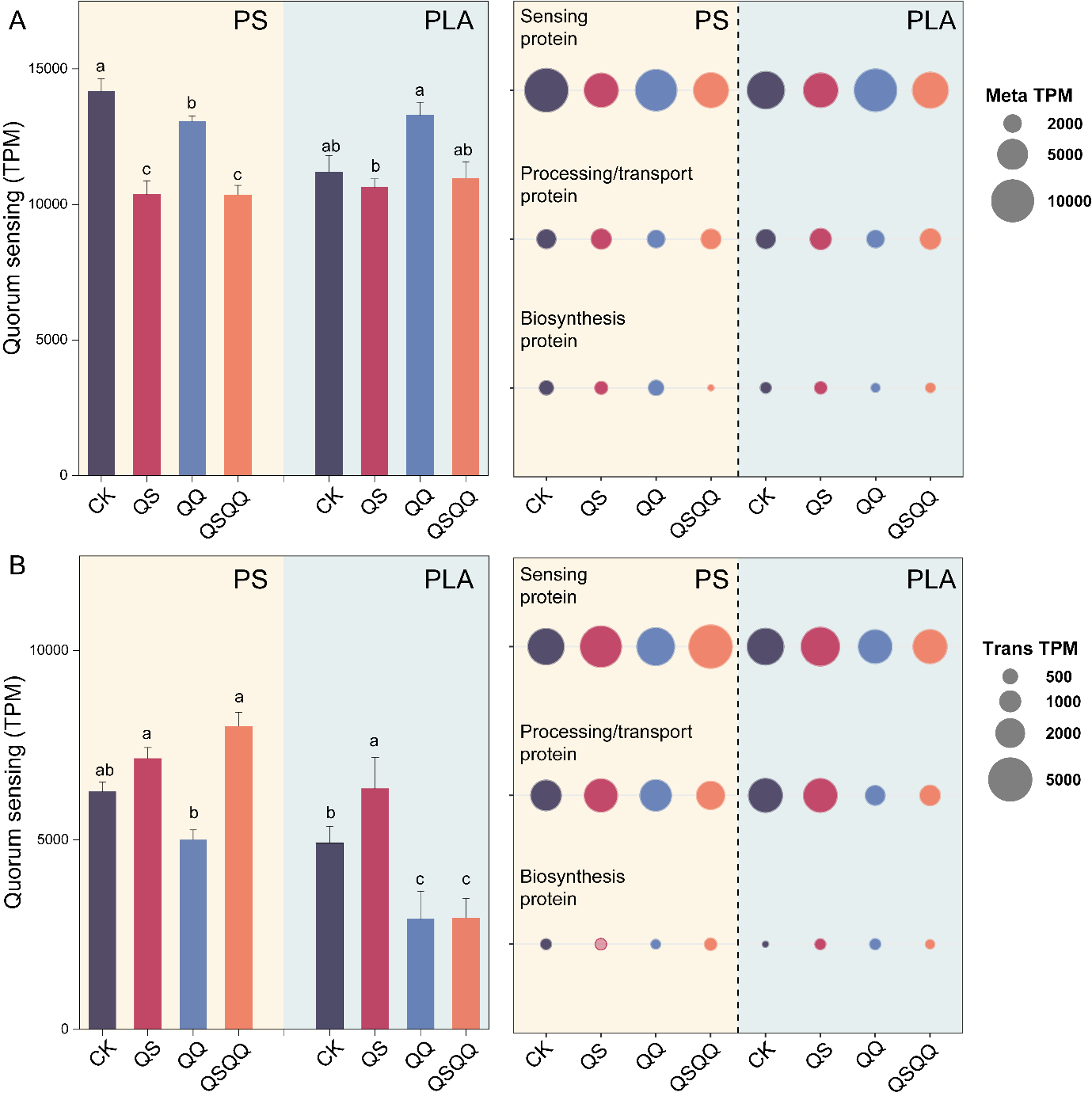


Figure S11. The gene abundance (A) and transcript activity (B) of quorum sensing processes in different treatment. The bubble diagram is the gene abundance and transcript activity of three functional categories involved in quorum sensing.


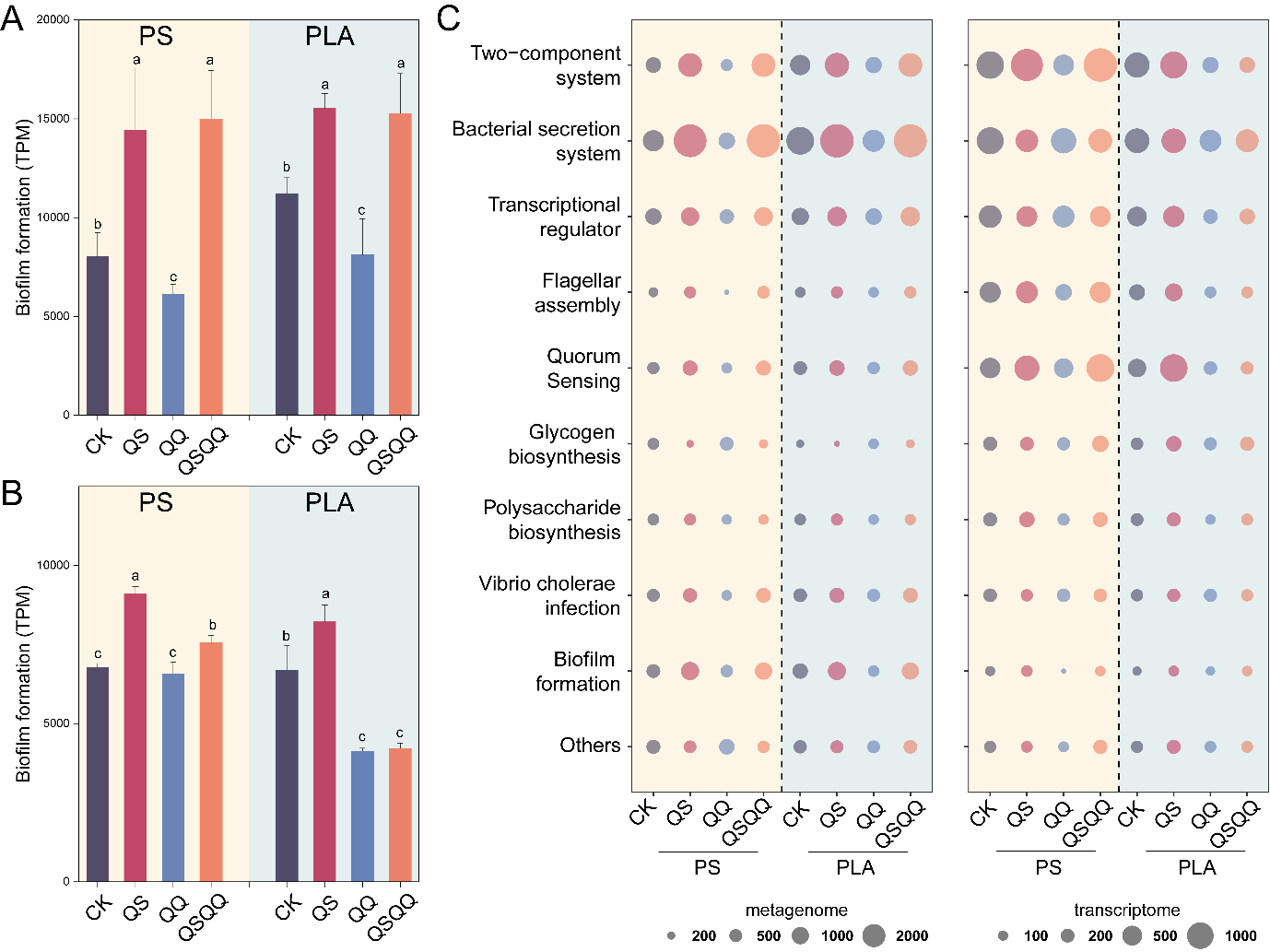


Figure S12. Biofilm formation pathway. The gene abundance (A) and transcript activity (B) of biofilm formation pathway in different treatment. The bubble diagram is the gene abundance and transcript activity of different functional categories involved in biofilm formation.


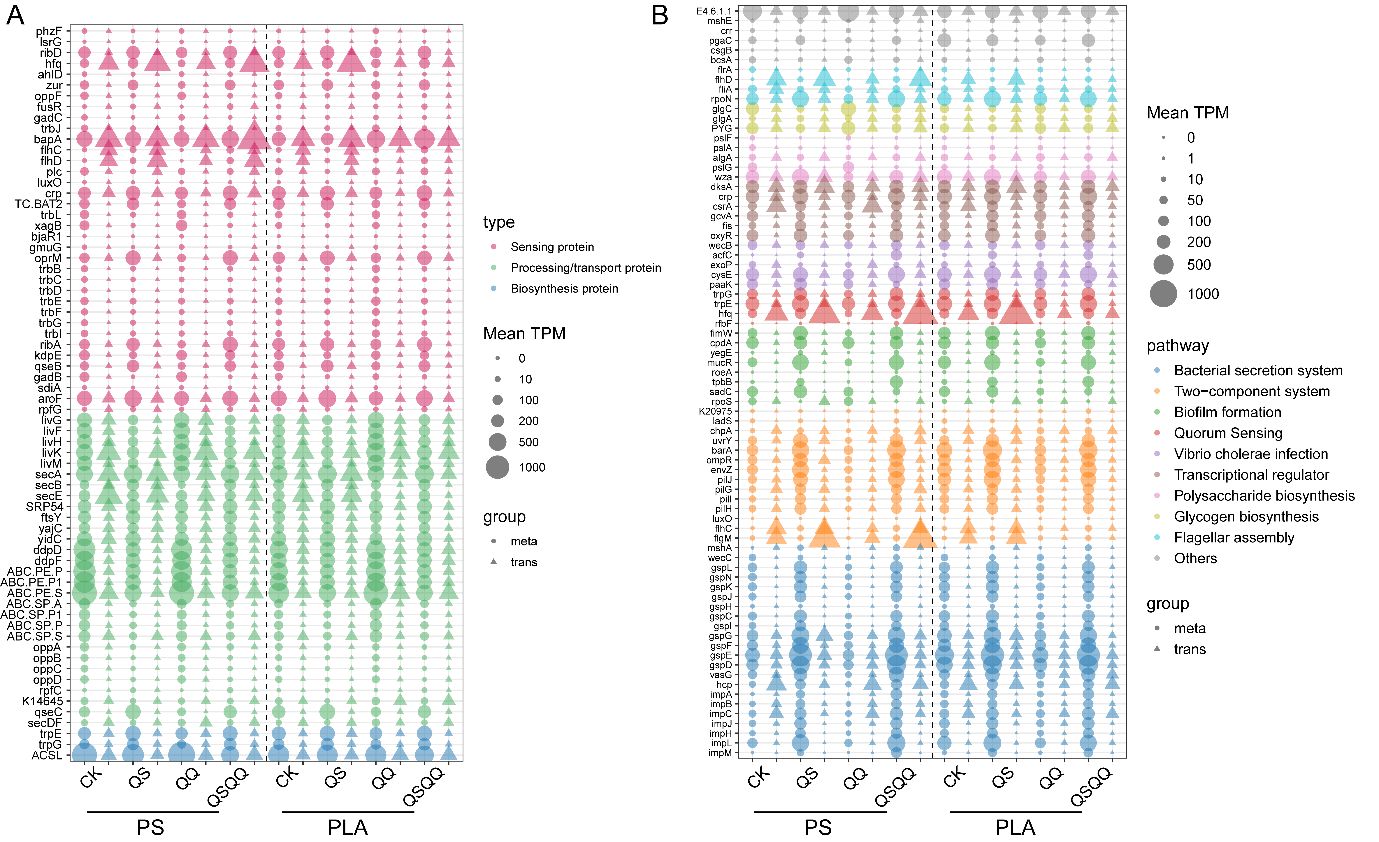


Figure S13. The gene abundance and activity in different treatments. A. Quorum sensing pathway; B. Biofilm formation pathway.


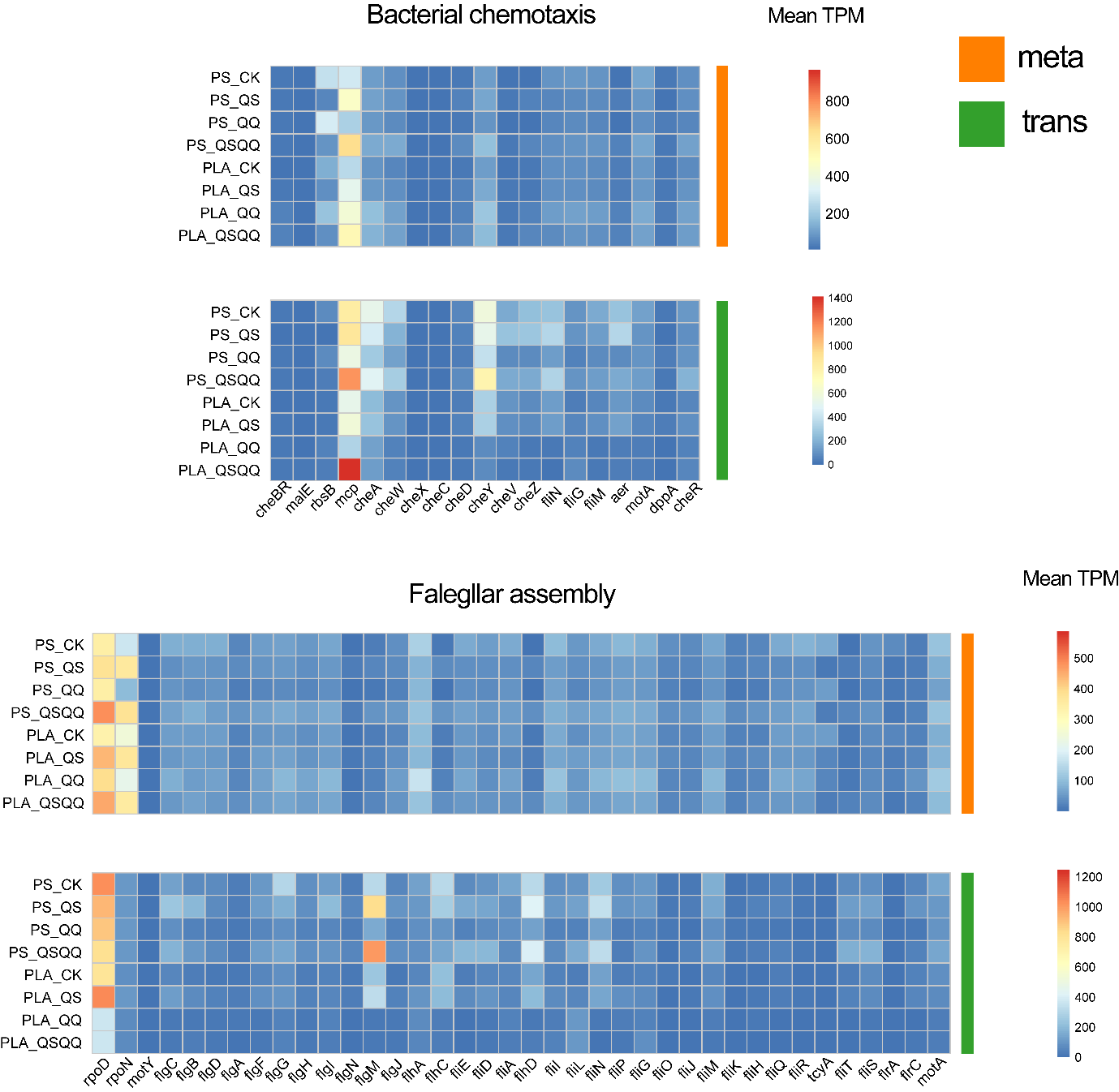


Figure S14. The gene abundance and activity in different treatments.


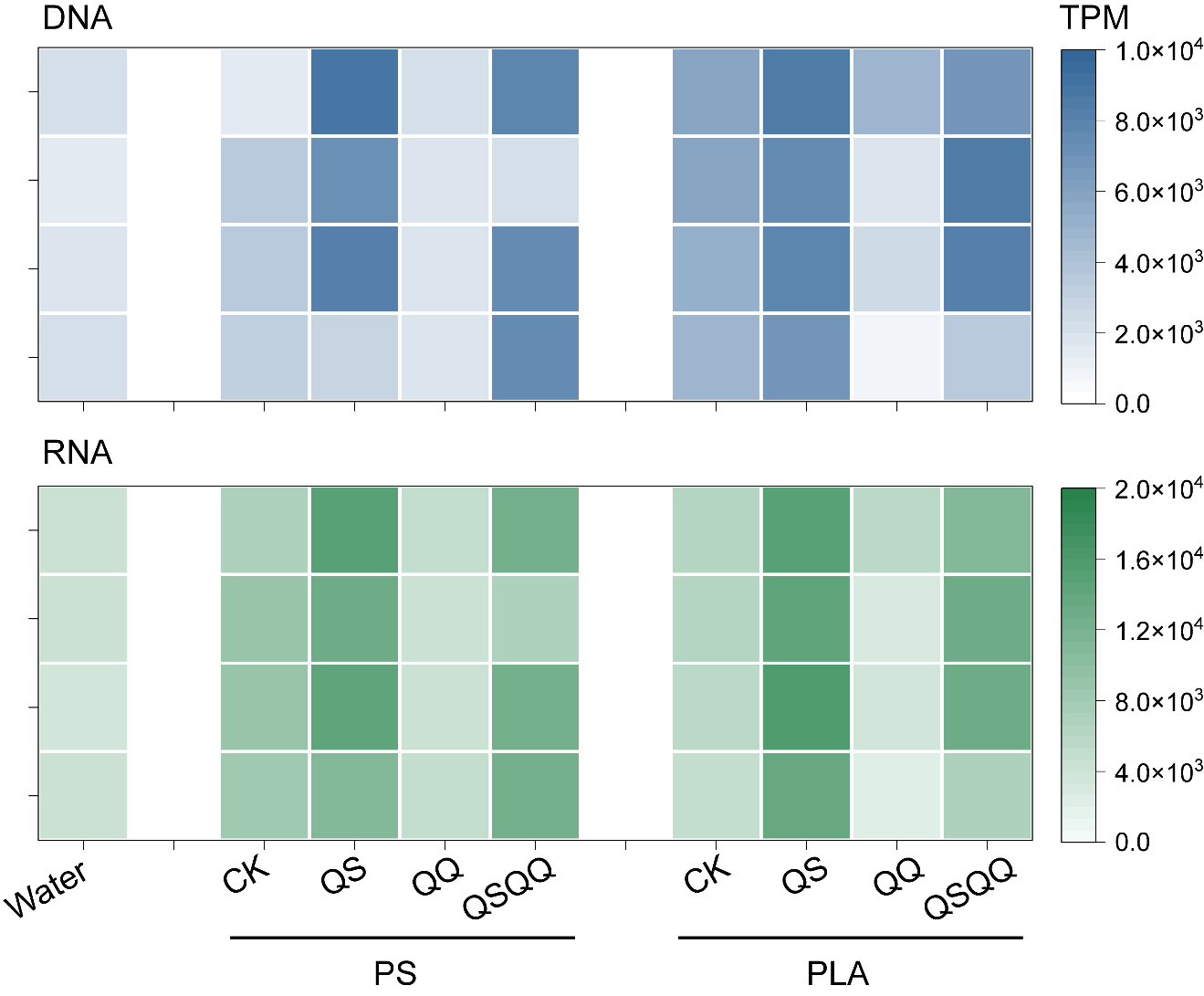


Figure S15. The abundance and activity of pathogens via metagenomics against Pathogen Host Interactions database.


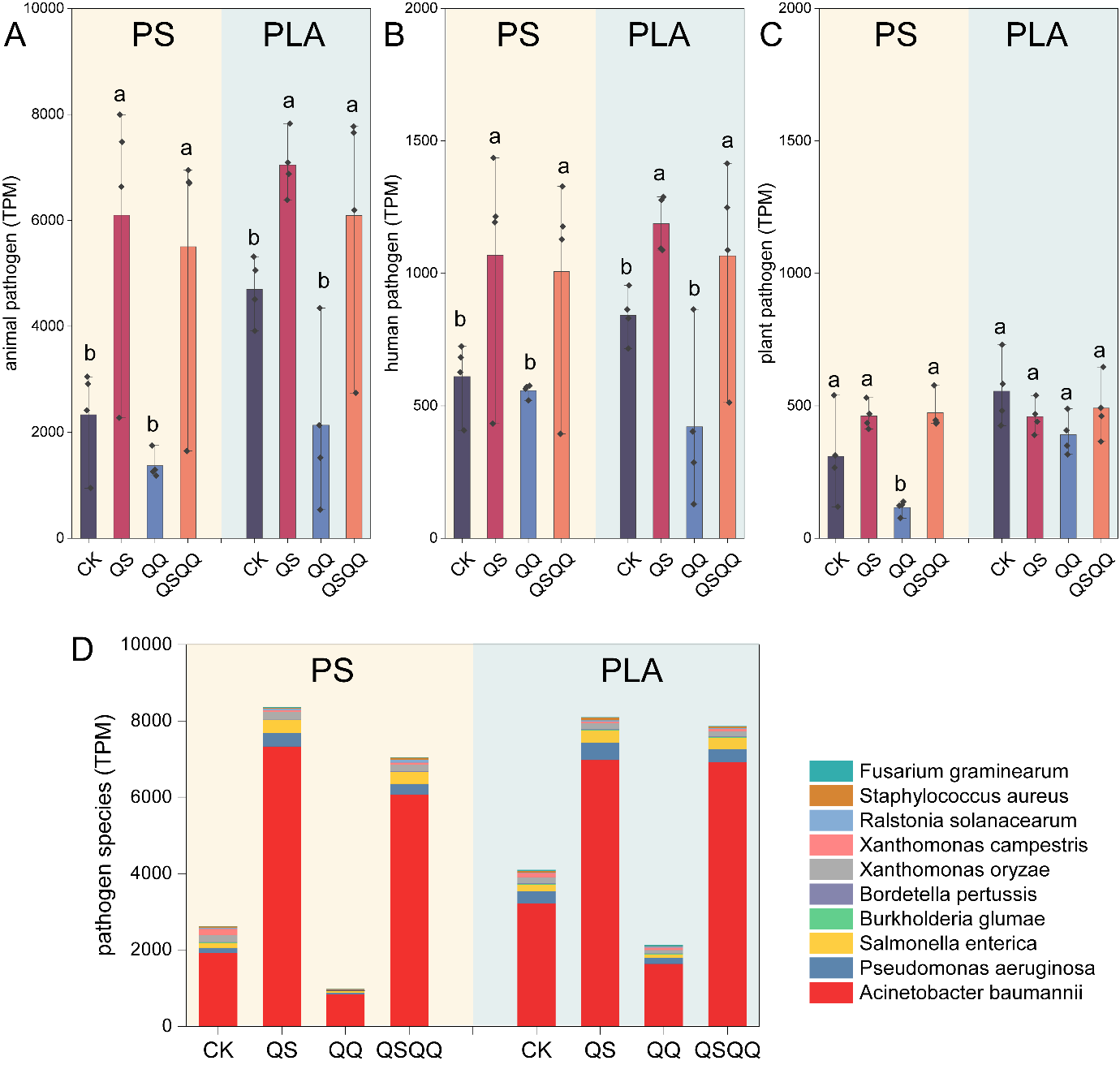


Figure S16. The pathogen identified via metagenomics against Pathogen Host Interactions database.


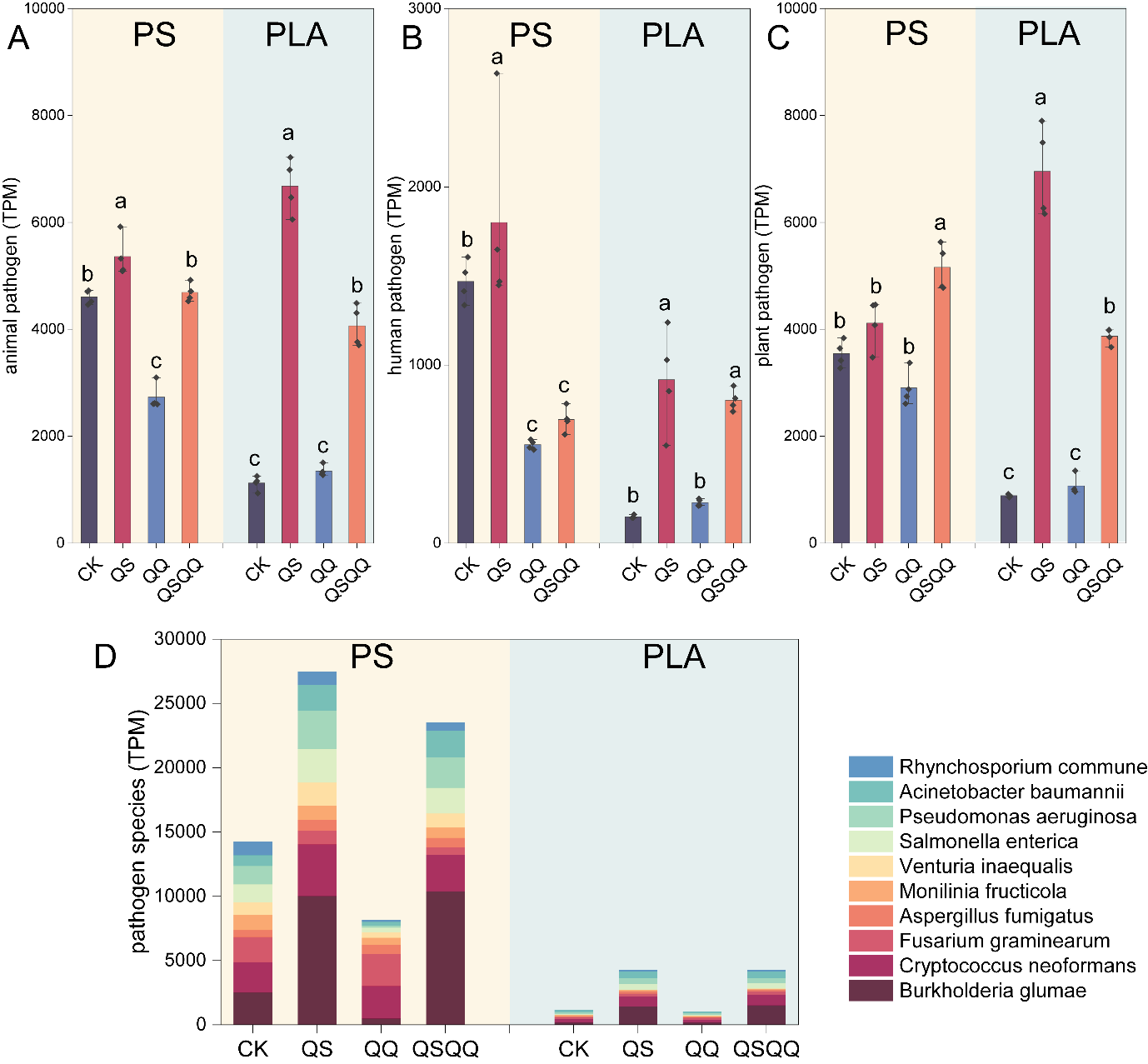


Figure S17. The pathogen identified via metatranscriptomic against Pathogen Host Interactions database.


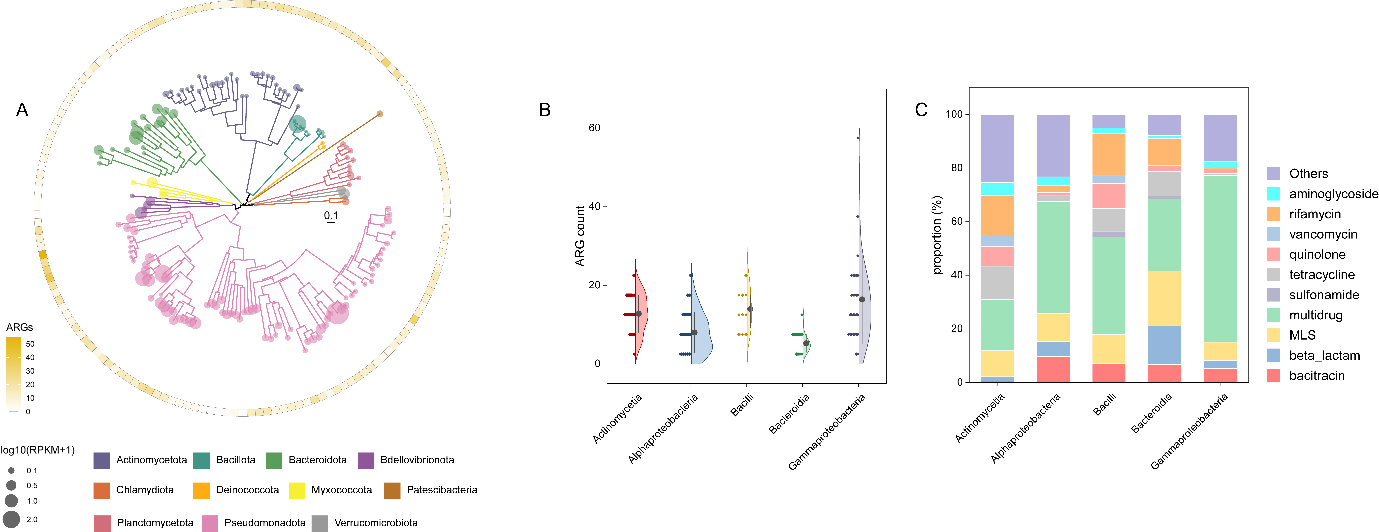


Figure S18. The ARGs on the metagenome-assembled genomes (MAGs). A. The phylogenetic tree of MAGs. The leaf colors indicate phylum groups. The yellow ring shows the ARG number in the MAGs. B. The count of ARGs across phylogenetic groups. C. The proportion of different ARG types across phylogenetic groups.


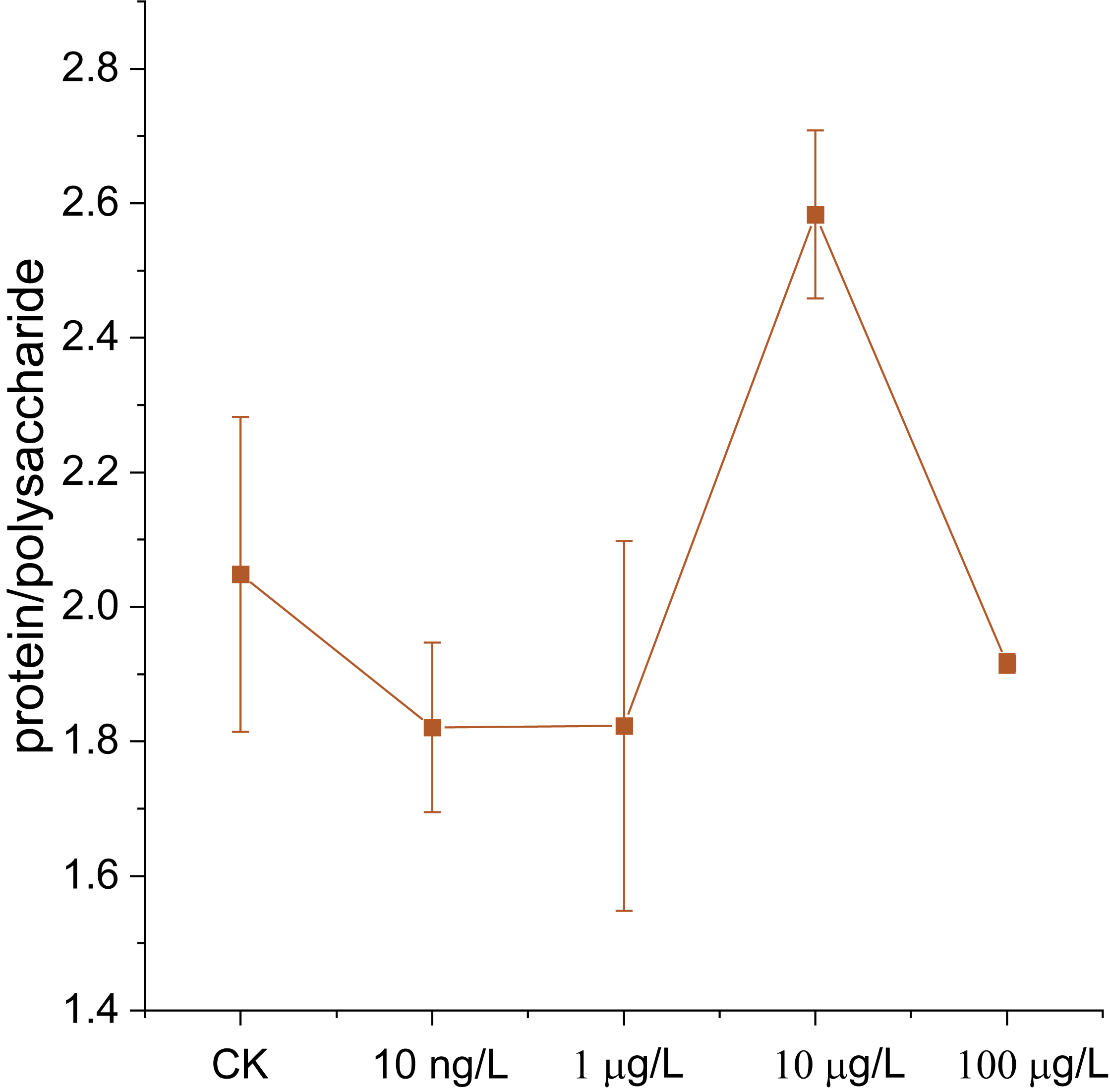


Figure S19. The ratio of protein to polysaccharide in EPS matrix under different concentration of 3OC6-HSL.


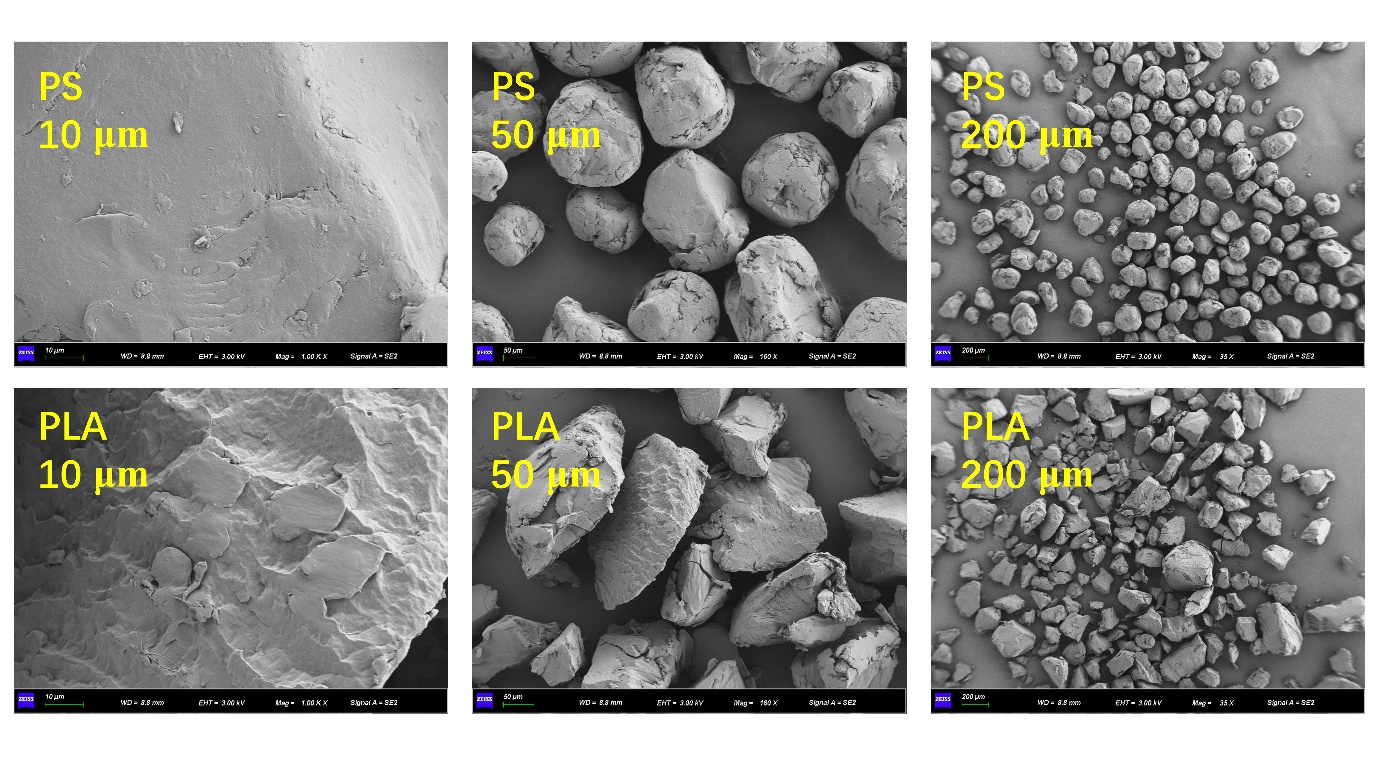
Figure S20. Scanning electron microscopy (SEM) micrographs of PS and PLA microplastics.
